# Supplementary material for: Geospatial Metabolomics Unravel Regional Disparities in Sedative Compounds and Volatile Profiles of Ziziphi Spinosae Semen Across Chinese Production Areas
Source: Plants (Basel). 2025 Sep 2;14(17):2739. doi: 10.3390/plants14172739 (PMC12430372; doi:10.3390/plants14172739)
Supplement: Supplementary file 1 [file plants-14-02739-s001.zip › plants-3808157-supplementary.pdf]

Table S1

Ten batches of sour jujube fruits collected from different geographical origin in China.				
Ziziphi Spinosae Semen (ZSS)				
**Sample**	**Source**	**region**	**Longitude–latitude**	**Climate characteristics**
GS-QY	Wildness	Qingyang, Gansu	35°42′N-107°38′E	Temperate continental climate; arid with low rainfall; abundant sunshine; mean annual temperature 9-11°C; annual precipitation 380-600 mm.
Sx-WN	Wildness	Weinan, Shaanxi	34°30′N-109°30′E	Warm temperate semi-humid monsoon climate; four distinct seasons; sufficient sunlight; mean annual temperature 12-14°C; annual precipitation ~600 mm.
SX-YC	Wildness	Yuncheng, Shanxi	35°20′N-110°59′E	Warm temperate continental monsoon climate; simultaneous rain and heat; mean annual temperature 12-13°C; annual precipitation 500-600 mm.
SX-CZ	Wildness	Changzhi, Shanxi	37°39′N-111°80′E	Warm temperate continental monsoon climate; mild winter and cool summer; large diurnal temperature variation; mean annual temperature 9-10°C; annual precipitation 550-650 mm.
SX-LL	Wildness	Lvliang, Shanxi	37°31′N-114°23′E	Temperate continental monsoon climate; cold winter and cool summer; mean annual temperature 7-9°C; annual precipitation 450-550 mm.
HB-CD	Wildness	Chengde, Hebei	42°10′N-118°42′E	Temperate continental monsoon climate; long winter and short summer; large diurnal temperature variation; mean annual temperature 7-9°C; annual precipitation 500-600 mm.
HB-ZH	Wildness	Zanhuang, Hebei	37°39′N-114°23′E	Warm temperate continental monsoon climate; dry and windy spring; hot and rainy summer; mean annual temperature 12-13°C; annual precipitation 500-600 mm.
HB-XT	Wildness	Xingtai, Hebei	37°40′N-114°30′E	Warm temperate continental monsoon climate; simultaneous rain and heat; mean annual temperature 13-14°C; annual precipitation 500-600 mm; concentrated summer rainfall.
SD-LY	Wildness	Linyi, Shandong	35°03′N-118°20′E	Warm temperate monsoon climate; simultaneous rain and heat; four distinct seasons; mean annual temperature 12.5-13.5°C; annual precipitation 800-900 mm.
LN-CY	Wildness	Chaoyang, Liaoning	42°01′N-121°39′E	Temperate continental monsoon climate; windy spring and autumn; cold winter and hot summer; annual temperature 8-9°C; annual precipitation 450-550 mm.

Table S2

Relative expression of metabolites in different regions												
	Class	Compounds	Sx-WN	SX-LL	HB-CD	LN-CY	SX-YC	GS-QY	SX-CZ	SD-LY	HB-ZH	HB-XT
1	Fatty acids and conjugates	3-(1-Hydroxymethyl-1-propenyl)pentanedioic acid	5.0796	5.0473	5.1261	5.2684	5.0031	5.1068	5.0045	5.1269	5.096	5.058
2	Fatty acids and conjugates	Glutarate semialdehyde	5.9384	5.8274	5.981	6.2274	6.0334	6.1337	5.9145	6.2584	6.2389	6.0892
3	Fatty acids and conjugates	Goshuyic acid	5.3847	5.4684	5.6195	5.3534	5.4004	5.3862	5.3991	5.3661	5.3307	5.4866
4	Fatty acids and conjugates	(9Z,11R,12S,13S,15Z)-12,13-Epoxy-11-hydroxy-9,15-octadecadienoic acid	6.2009	6.0461	6.3368	6.495	6.202	6.6896	6.3444	5.9727	5.9695	6.2453
5	Fatty acids and conjugates	Xi-5-Hydroxydecanoic acid	4.0177	4.188	4.1065	5.3951	4.5119	4.4275	4.197	3.589	3.516	3.9464
6	Fatty acids and	Myristic acid	3.9523	4.2403	4.0645	4.049	4.0274	3.924	4.0983	4.1264	4.0895	3.9738

	conjugates											
7	Fatty acids and conjugates	9,12,13-TriHOME	5.9396	5.5518	5.5776	6.0303	6.0877	6.3473	6.1775	5.5283	5.3058	5.9594
8	Fatty acids and conjugates	7-hexadecynoic acid	4.1574	4.1981	4.1718	4.3647	4.0417	3.8857	3.9613	3.7607	3.8481	3.9922
9	Fatty acids and conjugates	Tetradecanedioic acid	4.6653	4.7029	4.716	4.5532	4.7875	5.3558	4.6893	4.4133	4.4411	4.5225
10	Fatty acids and conjugates	ACEXAMIC ACID	6.254	6.1586	6.1878	6.2915	6.3953	6.5542	6.3118	6.0264	6.2663	6.1529
11	Fatty acids and conjugates	D-Dethiobiotin	4.6393	4.2611	4.3324	4.7046	4.4432	4.5885	4.6668	4.1754	3.966	4.1001
12	Fatty acids and conjugates	Dethiobiotin	2.5211	2.4302	4.6085	2.4688	2.4739	2.6315	2.5013	2.3909	2.4335	2.4496

13	Fatty acids and conjugates	10-hydroxy-(2E,8E)-decadien-4-ynoic Acid	4.734	4.1167	3.5024	3.5802	5.1499	4.1543	4.1758	3.292	2.8612	3.8538
14	Fatty acids and conjugates	Dioscoretine	4.7196	4.5875	4.4359	4.4954	5.3537	5.1545	4.7983	4.84	4.8589	5.3741
15	Fatty acids and conjugates	16-Hydroxy-10-oxohexadecanoic acid	3.4441	3.6693	5.3167	3.6815	3.7666	3.5219	3.6186	3.8197	3.7529	3.4497
16	Fatty acids and conjugates	3-Oxotetradecanoic acid	2.9817	2.8273	2.9222	4.4927	2.902	6.048	2.9948	3.7969	2.8826	3.4454
17	Fatty acids and conjugates	3,4-Dimethyl-5-propyl-2-furantridecanoic acid	4.6567	4.6443	3.1808	3.7169	4.9171	5.5205	4.7712	4.4756	4.8041	5.5178
18	Fatty acids and conjugates	FAHFA(16:0/9-O-18:0)	5.8786	4.174	4.6952	4.5934	5.2695	5.6676	5.1441	4.4377	4.5099	5.3677
19	Fatty acids and	3-Methyl-5-pentyl-2-furannonanoic acid	5.7613	5.8089	5.5366	5.5762	5.4212	6.5496	6.0086	5.073	5.4565	6.3551

	conjugates											
20	Fatty acids and conjugates	Docosahexaenoic acid	4.2831	4.1652	4.0796	4.2764	4.1855	4.1597	4.1667	3.9717	3.7741	4.1651
21	Fatty acids and conjugates	2-Hydroxyundecanoate	4.6383	4.6781	4.6176	4.727	4.6925	4.6939	4.6413	4.6811	4.6105	4.7423
22	Fatty acids and conjugates	Stearic acid	4.5943	3.1273	3.992	4.0567	4.2728	5.9416	2.9879	3.8541	3.8274	5.2056
23	Fatty acids and conjugates	Floionolic acid	6.3536	6.4197	6.2857	6.3771	6.337	6.387	6.3588	6.2666	6.2668	6.3507
24	Fatty acids and conjugates	Cerebronic acid	5.5987	5.9139	5.9337	5.7048	5.7057	5.7827	5.8439	5.6742	5.806	5.6731
25	Fatty acids and conjugates	4-Heptenoic acid	5.0761	5.1174	5.0411	5.141	5.1301	5.027	5.1091	5.1371	5.1239	5.1098

26	Fatty acids and conjugates	12(13)Ep-9-KODE	4.57	4.37	4.6933	4.7659	4.7802	5.3158	4.8111	4.4597	4.3485	4.6977
27	Fatty acids and conjugates	AUDA	5.1688	4.679	4.7506	4.7446	5.0161	5.5265	5.4928	4.2859	3.1148	4.1457
28	Fatty acids and conjugates	(10Z,14E,16E)-10,14,16-Octadecatrien-12-ynoic acid	5.3927	4.7833	4.9827	5.3513	5.8892	5.704	5.5867	4.705	3.8127	4.8065
29	Fatty acids and conjugates	Tetracosatetraenoic acid (24:4n-6)	4.3371	4.3319	5.5431	4.5574	4.3417	5.2237	4.637	3.975	4.5369	4.356
30	Fatty acids and conjugates	Pentadecanoic acid	2.8685	3.6746	3.6339	3.8801	4.0022	3.8443	3.149	2.9316	3.1608	2.8341
31	Fatty acids and conjugates	20-Carboxy-leukotriene B4	4.1202	4.0424	3.9075	4.5755	4.0632	4.149	3.9533	3.8986	3.8445	3.7245
32	Fatty acids and	2-Butyl-5-[2-(4-hydroxy-3-methoxyphenyl)ethyl]	4.4265	4.2091	4.2362	4.7762	4.2562	4.2315	4.2014	4.2059	4.1215	4.2766

	conjugates	furan										
33	Fatty acids and conjugates	2,4,7-Decatrienoic acid	3.1101	2.7426	2.8251	2.6144	2.8212	3.4136	3.188	2.9633	3.0373	3.4999
34	Fatty acids and conjugates	3-Isopropenylpentanedioic acid	5.2226	5.6606	5.2193	5.2784	5.2912	5.3949	5.6552	5.2328	5.4001	5.3657
35	Fatty acids and conjugates	3,4-Methyleneadipic acid	4.8153	2.6116	2.7293	2.702	3.2276	4.6569	3.7141	2.471	3.8026	4.83
36	Fatty acids and conjugates	(2E,4E)-2,7-Dimethyl-2,4-octadienedioic acid	5.0138	4.8857	5.0795	5.396	4.9678	5.2022	5.0786	4.9082	4.8089	4.7955
37	Fatty acids and conjugates	(Z)-10-Hydroxy-8-decene-4,6-diynoic acid	4.1958	3.1456	3.9791	3.8442	3.2912	3.5634	3.3515	3.1504	3.15	3.2647
38	Fatty acids and conjugates	3-Hydroxy-2-methylglutarate	3.1003	3.0784	3.6539	4.8134	3.1411	3.5982	3.1342	3.0579	3.0575	4.1428

39	Fatty acids and conjugates	1-Pentadecanecarboxylic acid	0.7581	0.127	0.1295	0.7972	0.1512	0.1623	0.1491	0.1282	0.6468	0.1441
40	Fatty acids and conjugates	2-Methyl-4-pentenoic acid	3.3731	3.34	3.5415	3.4227	3.3959	3.408	3.5008	3.3774	3.3307	3.3018
41	Fatty acids and conjugates	4-Formyl-3-(formylmethyl)-4-hexenoic acid	6.717	6.5834	6.2157	6.0767	6.6066	7.0075	6.5469	6.154	6.495	6.5255
42	Fatty acids and conjugates	2-hydroxyhexadecanoic acid	7.3189	7.0647	7.0985	7.4247	7.221	7.4548	7.1285	6.9356	6.7768	6.9301
43	Fatty acids and conjugates	Ethyladipic acid	5.2782	5.2037	5.5625	5.5667	5.3435	5.2919	5.4118	5.268	5.436	5.3178
44	Fatty acids and conjugates	3-Hydroxymethylglutaric acid	4.0961	4.1146	4.0654	4.9087	3.9927	4.0854	3.9907	3.8878	3.9873	4.0369
45	Fatty acids and	(Z)-3-(1-Formyl-1-propenyl)pentanedioic acid	3.9244	4.8645	4.5098	5.0391	4.2699	4.4366	4.9115	4.2327	4.6322	4.6143

	conjugates											
46	Fatty acids and conjugates	Suberic acid	5.596	5.4819	5.5706	5.6016	6.1519	6.3992	5.6269	5.6481	5.919	6.4266
47	Fatty acids and conjugates	2-Ethyl-2-Hydroxybutyric acid	5.7711	5.6001	5.6707	5.8835	5.7322	5.7504	5.726	5.5404	5.5038	5.4916
48	Fatty acids and conjugates	Caproic acid	4.9795	5.7359	5.5423	5.6669	4.7454	5.891	5.1906	5.253	4.3694	5.0259
49	Fatty acids and conjugates	Sebacic acid	5.6087	5.6262	5.5827	5.7248	5.691	6.1531	5.585	5.608	5.745	5.9947
50	Fatty acids and conjugates	3,4-Methylenesebacic acid	5.2765	5.2216	5.1621	5.2698	5.8062	5.654	5.3654	5.3275	5.3539	5.5717
51	Fatty acids and conjugates	12-hydroxyicosanoic acid	6.2258	5.906	6.0625	6.4162	6.1962	6.609	6.1416	6.0083	5.723	6.0245

52	Fatty acids and conjugates	9(10)-EpODE	5.263	5.3266	5.2788	5.3615	5.3288	5.4996	5.3736	5.3032	5.1831	5.2199
53	Fatty acids and conjugates	1,11-Undecanedicarboxylic acid	5.6667	5.7379	5.77	5.7053	5.679	5.6586	5.673	5.7559	5.8039	5.7124
54	Fatty acids and conjugates	3,4-Dimethyl-5-pentyl-2-furanpropanoic acid	4.5961	4.3055	4.7429	4.8007	4.6856	4.7776	4.5932	4.3698	4.5022	4.6757
55	Fatty acids and conjugates	Azelaic acid	4.4677	4.4633	4.6387	4.7464	4.9135	4.7397	4.4653	4.1888	4.1523	4.3923
56	Fatty acids and conjugates	Adipic acid	4.8674	5.0908	5.3914	5.1116	5.2652	5.0685	4.9634	4.826	4.8307	5.0399
57	Fatty acids and conjugates	CMPF	2.7682	2.8634	3.3556	3.7069	3.5759	3.5677	3.2713	3.1907	2.7246	3.3152
58	Fatty acids and	2-Ethylsuberic acid	4.0177	3.9527	3.9405	3.8059	4.5119	5.0444	3.9026	4.4012	4.355	5.0753

	conjugates											
59	Fatty acids and conjugates	Undecanoic acid	3.5428	3.3978	3.0917	3.5865	3.6352	4.0293	3.6927	3.034	3.5322	3.3776
60	Fatty acids and conjugates	Undecylenic acid	4.6031	4.514	5.0175	6.1283	4.6673	5.2539	4.7661	4.6492	4.4997	4.6148
61	Fatty acids and conjugates	11-Oxohexadecanoic acid	4.7113	4.6376	4.5278	4.7732	4.8448	5.1916	4.8206	4.5542	4.6711	4.5915
62	Fatty acids and conjugates	2,4-Dimethylpimelic acid	4.7733	4.5653	4.7381	4.3863	5.1374	4.9284	4.8449	5.1865	5.5417	5.4417
63	Fatty acids and conjugates	9,10-DHOME	3.1902	3.1872	3.2524	3.2815	3.7851	4.3813	3.4018	3.3514	4.1753	4.3244
64	Fatty acids and conjugates	12-hydroxyheptadecanoic acid	5.3463	6.1385	5.3884	5.4081	5.4604	5.8422	5.189	5.0344	5.3627	5.8952

65	Fatty acids and conjugates	5-Tetradecenoic acid	4.6037	4.6937	4.8112	4.7536	4.741	4.8153	4.6314	4.71	4.7531	4.7476
66	Fatty acids and conjugates	6-Hydroxypentadecanedioic acid	4.2472	4.1479	4.4556	4.7041	4.3216	4.0236	4.7709	4.0776	4.1851	4.1847
67	Fatty acids and conjugates	MEDICA 16	5.0285	4.7995	4.9066	5.0763	5.1309	5.6668	4.8109	4.9153	5.1464	5.5848
68	Fatty acids and conjugates	Hypogeic acid	4.0942	4.1307	4.1064	3.895	4.274	5.1901	3.2858	4.3897	4.2939	4.5315
69	Fatty acids and conjugates	3-hydroxypentadecanoic acid	5.1677	5.0527	5.0145	5.2595	5.2359	5.4038	5.2675	5.1989	4.8837	4.8829
70	Fatty acids and conjugates	3-Oxooctadecanoic acid	3.4855	3.2348	3.3232	3.8504	3.712	4.588	3.3733	3.2773	3.5128	3.8162
71	Fatty acids and	9,10,13-TriHOME	3.7831	3.7092	3.3746	3.6769	3.6966	3.9864	3.9203	3.5128	3.1895	3.4345

	conjugates											
72	Fatty acids and conjugates	(9S,10S)-9,10-dihydroxy octadecanoate	4.6071	3.9347	3.9142	4.1919	4.2169	4.4949	4.0765	3.8957	4.1088	4.6693
73	Fatty acids and conjugates	7Z,10Z-Hexadecadienoic acid	4.0037	4.4696	4.1945	4.2158	4.3713	4.5421	4.6275	4.0022	4.3082	4.1908
74	Fatty acids and conjugates	Ganosporeric acid A	5.8004	4.125	3.1864	3.8379	3.4044	5.1104	3.2174	3.648	3.1467	4.5187
75	Fatty acids and conjugates	Tetranor 12-HETE	4.8246	4.3497	4.1944	4.4295	5.4602	5.5472	5.2768	4.6828	4.2726	4.6351
76	Fatty acids and conjugates	(R)-2-Hydroxycaprylic acid	2.5918	3.3179	2.7357	2.7371	2.7951	2.7613	3.0157	3.0537	3.4265	3.338
77	Fatty acids and conjugates	Docosatrienoic acid	4.0289	4.0298	4.0805	4.7283	4.2054	3.6348	3.692	4.4433	2.0714	2.9459

78	Fatty acids and conjugates	2-Hydroxymyristic Acid	5.2442	5.0766	5.0448	5.5955	5.5132	4.9888	5.2126	5.1043	5.0251	5.0298
79	Fatty acids and conjugates	10,20-Dihydroxyeicosanoic acid	5.9148	5.5214	5.5976	5.7103	5.5088	6.0517	5.5279	5.5006	5.5889	6.2164
80	Fatty acids and conjugates	3-Methyl-5-propyl-2-furantridecanoic acid	4.3981	4.7865	4.496	4.6437	4.4759	4.2784	4.4436	4.6801	4.0952	4.0239
81	Fatty acids and conjugates	(Z)-15-Oxo-11-eicosenoic acid	4.147	4.1174	4.0482	3.1872	4.2084	4.1921	4.2797	4.0008	3.7828	4.4741
82	Fatty acids and conjugates	(S)-10,16-Dihydroxyhexadecanoic acid	5.7106	5.3845	5.2577	5.4666	5.3974	5.5238	5.5014	5.3491	5.3009	5.4594
83	Fatty acids and conjugates	Tridecanoic acid	4.7979	4.6423	4.5638	4.5758	5.0554	4.7282	4.8004	4.4739	4.5473	4.6413
84	Fatty acids and	Monic acid	4.3466	4.3936	4.9847	4.556	4.7298	5.1213	4.6076	4.6679	5.0474	5.2017

	conjugates											
85	Fatty acids and conjugates	9,10,13-Trihydroxystearic acid	5.6445	5.6913	5.71	5.9152	5.5518	5.9741	5.7996	5.5629	5.5983	5.7249
86	Fatty acids and conjugates	9-Oxo-nonanoic acid	5.9612	5.733	5.9091	6.1994	6.0773	6.2636	6.0748	5.8048	5.9793	6.2119
87	Fatty acids and conjugates	11-Hydroxy-9-tridecenoic acid	6.5053	6.2116	6.1496	6.4017	6.7747	6.737	6.7131	5.9996	6.0622	6.1029
88	Fatty acids and conjugates	3-carboxy-4-methyl-5-pentyl-2-furanpropanoic acid	4.3223	4.1532	4.2494	4.5222	4.5774	4.6754	4.3335	4.0851	3.807	4.3079
89	Fatty acids and conjugates	11Z-Eicosenoic acid	4.0568	3.8519	4.8255	4.8503	3.8981	5.3557	4.423	4.3908	3.139	4.05
90	Fatty acids and conjugates	2-Ethylglutaric acid	4.2611	4.1979	3.8044	3.8231	3.897	4.8763	4.0463	3.9722	4.4751	4.6568

91	Fatty acids and conjugates	Macrophorin D	5.2272	5.1811	5.6288	5.4427	5.1795	5.2821	5.409	5.2586	4.8883	5.1359
92	Fatty acids and conjugates	Undecanedioic acid	4.6207	4.7177	4.9976	4.9864	4.9664	5.0867	4.8035	4.6505	4.9253	5.4409
93	Fatty acids and conjugates	2-Hydroxycaproic acid	3.3994	3.3289	3.3292	3.5114	3.3505	3.4253	3.3094	3.3031	3.3125	3.3706
94	Fatty acids and conjugates	Traumatic acid	4.4134	4.3143	4.5819	4.9442	4.3614	4.3999	4.4218	4.3667	4.2636	4.3613
95	Fatty acids and conjugates	Heptanoic acid	3.319	3.3444	3.6596	3.5384	3.9039	3.6142	3.5968	3.5028	3.5586	3.3985
96	Fatty acids and conjugates	2-Octenedioic acid	4.931	2.6074	4.2083	2.4369	2.9283	5.1143	3.8423	2.8628	3.8144	4.3505
97	Fatty acids and	Wyeronic acid	4.0253	4.758	4.6583	3.9457	4.5412	4.6324	4.6627	4.6266	4.9261	4.5586

	conjugates											
98	Fatty acids and conjugates	6-(2-Hydroxyethoxy)-6-oxohexanoic acid	4.6714	5.0823	4.7899	4.8688	4.6645	4.7908	5.1258	4.5188	4.8127	4.9348
99	Fatty acids and conjugates	Mono-methyl-adipate	4.7177	4.6526	4.6792	5.0817	4.7058	4.3965	4.7225	4.6838	4.7174	4.9628
100	Fatty acids and conjugates	Mevalonic acid	5.6072	5.4383	5.641	5.8984	5.4392	5.6575	5.504	5.4175	5.4396	5.475
101	Fatty acids and conjugates	2-Propylglutaric acid	3.3892	3.4151	3.4702	3.5712	3.396	3.318	3.4047	3.49	3.4285	3.5338
102	Fatty acids and conjugates	13-Oxo-9,11-tridecadienoic acid	3.9018	3.4441	3.8227	4.0272	3.8402	3.8048	3.8295	3.6277	3.4402	3.8666
103	Fatty acids and conjugates	3-Methyladipic acid	4.842	4.8131	5.0053	5.0049	4.8949	4.854	4.8226	4.8611	4.6966	4.6897

104	Fatty acids and conjugates	8-[(Aminomethyl)sulfanyl]-6-sulfanyloctanoic acid	5.3462	4.7684	5.1629	5.3434	5.0457	5.4393	5.2803	4.8477	4.5062	5.0791
105	Fatty acids and conjugates	(+/-)-2-Hydroxy-4-(methylthio)butanoic acid	4.2745	3.8911	4.5081	4.8595	4.1735	4.0693	4.0847	4.071	3.6365	4.0166
106	Fatty acids and conjugates	Wyerone	5.814	5.362	5.1767	4.7064	4.9696	5.5613	4.9597	4.4309	4.4161	5.6089
107	Lineolic acids and derivatives	Mangiferic acid	6.0432	6.1148	5.933	6.136	6.1871	6.2401	6.3831	6.1469	6.1246	6.2653
108	Lineolic acids and derivatives	DG(18:0/18:2(9Z,12Z)/0:0)	6.7476	6.768	6.5928	6.6865	6.7285	6.654	6.639	6.7075	6.6519	6.6326
109	Lineolic acids and derivatives	MG(18:4(6Z,9Z,12Z,15Z)/0:0/0:0)	5.1218	4.9492	4.9844	4.9035	5.2254	5.2637	5.2824	5.3603	5.2723	5.4066
110	Lineolic acids and	MG(18:2(9Z,12Z)/0:0/0:0)[rac]	5.9807	5.548	5.5298	5.6783	5.6074	5.6074	5.6295	5.5568	5.5752	5.4766

	derivatives											
111	Lineolic acids and derivatives	DG(15:0/18:2(9Z,12Z)/0:0)	4.7837	4.515	4.635	4.5979	4.9952	5.7504	4.7736	4.9103	5.0526	5.5599
112	Lineolic acids and derivatives	DG(18:3(9Z,12Z,15Z)/18:1(11Z)/0:0)	6.2636	6.0414	5.9824	6.6308	6.1132	6.0575	6.0177	6.0359	5.8561	5.9395
113	Lineolic acids and derivatives	Calendic acid	4.0998	4.3726	4.0426	4.0843	4.0408	4.3241	3.9962	3.9234	3.8083	4.1075
114	Lineolic acids and derivatives	DG(15:0/18:4(6Z,9Z,12Z,15Z)/0:0)	5.7721	5.7988	5.6211	5.372	5.4634	6.1479	6.1408	4.795	5.1414	6.1701
115	Lineolic acids and derivatives	Bovinic acid	5.965	6.1626	6.1777	6.0057	6.1535	6.0549	5.9809	5.8173	5.8738	5.8804
116	Lineolic acids and derivatives	Dihomolinoleic acid	5.8479	5.5505	5.6047	5.8337	5.9275	5.5955	5.6571	5.6298	5.1817	5.5872

117	Lineolic acids and derivatives	10-Nitrolinoleic acid	4.5907	4.7829	4.9929	5.0297	4.9702	5.2628	4.9799	4.7183	4.9336	5.3253
118	Lineolic acids and derivatives	Avenoleic acid	5.7179	5.469	5.6801	5.7567	5.4683	5.7355	5.4631	5.2848	5.1145	5.2706
119	Lineolic acids and derivatives	DG(18:1(11Z)/18:2(9Z,12Z)/0:0)	6.1847	6.1602	6.0662	6.1687	6.1087	6.0854	6.0768	6.0392	5.938	6.0002
120	Lineolic acids and derivatives	DG(18:0/18:3(9Z,12Z,15Z)/0:0)	6.4996	6.5417	6.4342	6.4647	6.486	6.478	6.4745	6.4614	6.5124	6.4568
121	Lineolic acids and derivatives	DG(18:4(6Z,9Z,12Z,15Z)/18:2(9Z,12Z)/0:0)	5.0258	4.6883	3.3503	4.7892	4.7915	4.7154	4.5486	4.3555	4.1464	4.5823
122	Lineolic acids and derivatives	DG(18:2(9Z,12Z)/18:3(9Z,12Z,15Z)/0:0)	5.8256	5.5292	5.0645	5.9469	5.7139	5.5379	5.5676	5.4474	5.2018	5.2049
123	Lineolic acids and	DG(18:2(9Z,12Z)/16:0/0:0)	7.3474	7.3975	7.3051	7.3566	7.3983	7.3726	7.33	7.3697	7.3286	7.2761

	derivatives											
124	Lineolic acids and derivatives	DG(16:0/18:2(9Z,12Z)/0:0)	6.1554	6.3488	6.2576	6.0409	6.3304	6.3118	6.3091	6.4443	6.322	6.1788
125	Lineolic acids and derivatives	MG(0:0/18:3(6Z,9Z,12Z)/0:0)	4.6496	4.316	3.9918	4.4889	4.4513	4.1533	4.2615	4.0427	4.1786	4.2814
126	Lineolic acids and derivatives	Methyl linoleate	4.9225	4.3441	3.6643	4.4264	4.3311	5.6928	4.4494	3.8441	4.0795	5.3939
127	Lineolic acids and derivatives	DG(16:0/18:3(9Z,12Z,15Z)/0:0)	5.0724	4.0843	4.4831	5.0314	4.6493	4.5272	4.1956	4.3169	2.1099	4.3458
128	Lineolic acids and derivatives	DG(18:1(11Z)/18:3(9Z,12Z,15Z)/0:0)	6.6425	6.0077	5.9942	6.0673	6.1989	6.0428	6.0504	6.0577	5.9489	6.0376
129	Lineolic acids and derivatives	Alpha-dimorphecolic acid	5.669	5.7625	5.6834	5.4763	5.3179	5.9243	5.9485	4.8339	4.9965	5.7381

130	Lineolic acids and derivatives	DG(18:4(6Z,9Z,12Z,15Z)/16:1(9Z)/0:0)	4.528	3.9027	1.4192	3.9901	4.6971	5.1275	3.6322	4.0078	3.9013	5.9346
131	Lineolic acids and derivatives	Alpha-Linolenic acid	4.2788	5.3502	2.9647	4.6079	4.832	5.3742	5.3403	3.5821	2.7852	5.954
132	Lineolic acids and derivatives	8(R)-Hydroperoxylinoleic acid	5.2638	5.2685	5.1703	5.2079	5.2675	5.1885	5.1615	5.1869	5.1789	5.2034
133	Lineolic acids and derivatives	Pinolenic Acid	6.264	6.1258	6.0759	6.1289	6.2363	6.4189	6.2176	5.937	6.0142	6.45
134	Lineolic acids and derivatives	Jasmonic acid	4.1374	4.1837	4.5905	5.3714	4.7326	4.7407	4.6546	3.7133	3.311	3.9456
135	Lineolic acids and derivatives	Corchorifatty acid F	6.7066	6.6618	6.6309	6.7861	6.7095	6.9984	6.8589	6.532	6.7107	6.7292
136	Lineolic acids and	13(S)-HpODE	6.8923	6.8324	6.793	6.9462	6.9155	7.0177	6.8905	6.7773	6.7723	6.9303

	derivatives										
137	Lineolic acids and derivatives	9,10-DiHODE	6.2245	5.9108	5.9348	6.3278	6.0796	6.0817	6.0878	5.8817	5.7777	5.8357
138	Lineolic acids and derivatives	13-Hydroxy-9-methoxy-10-oxo-11-octadecenoic acid	3.6113	3.0858	3.1291	4.0098	4.0331	3.2569	3.2182	3.0867	3.1712	3.4061
139	Lineolic acids and derivatives	(9Z,12Z,14E)-16-Hydroxy-9,12,14-octadecatrienoic acid	4.9094	4.6751	4.7109	4.977	5.2239	4.9664	4.8429	4.6371	4.5051	4.7591
140	Lineolic acids and derivatives	Cucurbic acid	4.0128	3.8767	4.0901	3.8151	3.9151	4.1179	3.9023	3.8903	3.9293	4.529
141	Lineolic acids and derivatives	Dihydrojasmonic acid	5.2075	5.1561	5.0979	5.3556	5.1775	5.7595	5.4862	4.9681	4.9599	5.086
142	Lineolic acids and derivatives	Corchorifatty acid A	3.1484	3.4351	3.6716	3.3122	3.5156	2.9982	3.3892	3.8913	3.7867	4.0681

143	Lineolic acids and derivatives	9(S)-HODE	6.7827	6.5444	6.4262	6.6648	6.564	6.7602	6.6368	6.4765	6.4502	6.6728
144	Lineolic acids and derivatives	DG(18:2(9Z,12Z)/15:0/0:0)	6.5172	6.5974	6.278	6.4504	6.2331	7.0852	6.5488	6.3459	6.3094	6.9829
145	Lineolic acids and derivatives	DG(15:0/18:3(6Z,9Z,12Z)/0:0)	6.5206	6.5888	6.4119	6.662	6.247	6.9609	6.5946	6.1423	6.1824	6.8789
146	Lineolic acids and derivatives	DG(15:0/18:3(9Z,12Z,15Z)/0:0)	5.8425	6.0081	5.8272	5.9816	5.549	6.161	5.9787	5.416	5.5416	6.1707
147	Lineolic acids and derivatives	FAHFA(22:6(4Z,7Z,10Z,13Z,16Z,19Z)/13-O-18:2(9Z,11E))	5.4662	6.0346	5.7187	5.5025	5.527	5.9276	6.1793	5.1316	5.12	5.62
148	Lineolic acids and derivatives	Cibaric acid	5.1211	5.2343	5.2236	5.2483	5.2949	5.0768	5.2756	5.0983	5.1837	5.1067
149	Lineolic acids and	(+/-)-(E)-13-Hydroxy-10-oxo-11-octadecenoic	3.6513	3.9018	4.1508	4.0302	4.0053	4.1679	3.8516	3.5276	3.8414	3.9542

	derivatives	acid									
150	Lineolic acids and derivatives	14-HDoHE	4.733	3.496	2.4143	2.4398	2.4362	5.6318	2.7348	2.3591	5.5566	3.8783
151	Lineolic acids and derivatives	MG(P-18:0e/0:0/0:0)	4.2527	4.4272	4.5902	4.864	4.5507	4.6206	4.5864	4.4533	3.9236	4.277
152	Eicosanoids	PGF2a ethanolamide	5.8839	5.4016	5.5057	5.5872	5.7417	5.5812	5.4404	5.7341	5.2741	5.6377
153	Eicosanoids	11R-HEPE	4.9973	5.0704	5.5091	5.099	5.2529	6.7459	5.5533	5.0086	4.906	5.3902
154	Eicosanoids	Prostaglandin PGE2 1-glyceryl ester	5.5433	5.6774	5.4058	5.3214	5.746	6.3786	6.159	5.4705	5.5185	5.8701
155	Eicosanoids	5,6-DHET	5.4115	5.6245	5.8028	5.5023	5.63	5.5251	5.6092	5.4131	5.542	5.4959
156	Eicosanoids	13,14-Dihydro PGE1	5.475	5.5006	5.4207	5.5861	5.5801	5.5122	5.5538	5.5505	5.5181	5.5954
157	Eicosanoids	17-phenyl-18,19,20-trinor-prostaglandin E2	4.8296	3.2541	3.2636	3.8399	3.442	3.3791	3.9533	3.2589	3.2585	3.3177
158	Eicosanoids	Leukotriene B5	4.9256	4.916	4.885	5.1854	4.8028	5.1315	4.6076	4.7544	4.9448	4.5294
159	Eicosanoids	Prostaglandin F1a	5.4398	5.1207	5.0722	5.6347	5.4264	5.471	5.3773	5.0859	5.0209	5.2015

160	Eicosanoids	8,9-DiHETrE	4.6139	4.6671	4.5474	4.6033	4.6722	4.6313	4.5642	4.5336	4.5147	4.5859
161	Eicosanoids	DG(18:2n6/0:0/20:5n3)	4.9225	3.6789	3.5977	4.8863	4.3887	4.3021	3.8325	3.6325	3.5743	4.4757
162	Eicosanoids	(13E)-11a-Hydroxy-9,15-dioxoprost-13-enoic acid	5.5596	5.6066	5.5115	5.5908	5.5751	5.4918	5.5672	5.6089	5.6118	5.549
163	Eicosanoids	11-deoxy-PGE1	4.2744	3.8321	4.6039	4.818	4.6482	5.1662	4.2754	3.6628	4.2128	5.0416
164	Eicosanoids	Leukotriene E3	6.3438	6.4518	6.313	6.4886	6.3715	6.1622	6.3295	6.3805	6.4154	6.3857
165	Eicosanoids	PGF2alpha-EA	5.4682	4.2758	3.7626	4.4957	4.7342	5.3988	5.1201	3.3372	3.3255	4.1386
166	Eicosanoids	12-Oxo-2,3-dinor-10,15-phytodienoic acid	4.6785	4.6742	4.847	4.6777	4.8883	4.8695	4.8017	4.5784	4.5445	4.6402
167	Eicosanoids	8-iso-15-keto-PGE2	5.0779	5.0803	5.3599	5.4719	5.1906	5.0993	5.1502	5.298	5.3891	5.0719
168	Eicosanoids	5(S),14(R)-Lipoxin B4	4.4155	2.8147	2.1422	2.2152	2.2203	5.3587	2.2134	2.1376	5.6245	4.0598
169	Eicosanoids	9-Deoxy-delta12-PGD2	5.4223	5.3399	5.1819	5.7284	5.0382	5.4021	5.2882	4.9175	5.119	5.6354
170	Eicosanoids	Leukotriene D5	5.2768	3.9671	5.5389	5.3166	4.992	4.6707	4.6328	5.5934	3.9306	4.9737
171	Eicosanoids	Leukotriene F4	4.4643	5.1169	4.4041	3.8498	4.2838	4.8638	4.6684	3.0291	5.4477	5.5794
172	Eicosanoi	12-Oxo-20-trihydroxy-le	3.7335	3.8777	4.0289	4.0629	2.9085	4.0387	3.6912	5.1661	3.7573	3.8625

	ds	ukotriene B4										
173	Eicosanoids	2,3-Dinor-6-keto-prostaglandin F1 a	4.8605	4.018	4.5336	3.8402	4.3503	4.6073	4.549	4.2516	3.6587	4.387
174	Eicosanoids	2,3-dinor, 6-keto-PGF1alpha	5.0801	5.1773	5.5067	5.4009	5.3039	5.693	5.4319	5.1364	5.2785	5.413
175	Eicosanoids	Thromboxane B2	4.3377	3.8451	3.7607	3.6719	4.0783	4.6047	4.8147	3.374	4.2587	4.2202
176	Eicosanoids	9S-hydroxy-11,15-dioxo-5Z,13E-prostadienoic acid	3.4261	2.7046	3.0579	3.1414	3.6132	3.638	3.0537	3.3803	3.7825	3.7562
177	Eicosanoids	13,14-Dihydro PGF-1a	5.2622	4.0375	3.2589	3.8726	4.77	4.7839	5.0362	3.9877	4.1007	4.7168
178	Eicosanoids	5-Hexyltetrahydro-2-furanoctanoic acid	5.4595	5.1301	5.6603	5.7012	5.3715	5.7891	5.3847	5.3326	5.4281	5.4137
179	Eicosanoids	Prostaglandin E2	5.8576	5.5459	5.2578	5.8442	5.7126	5.8517	5.697	5.4118	5.1456	5.4947
180	Eicosanoids	5(6)-Epoxy Prostaglandin E1	3.1297	3.5456	5.2596	5.5464	3.9701	3.9899	3.7267	3.9813	4.0235	3.9463
181	Eicosanoids	10,11-dihydro-20-trihydroxy-leukotriene B4	4.9191	4.7889	5.0031	4.7017	5.2669	4.8705	4.7451	5.4729	5.4367	5.7137
182	Eicosanoids	11-Dehydro-thromboxane B2	3.7849	3.5241	4.4622	4.1609	3.5409	5.3475	4.6247	3.688	3.0825	4.138
183	Eicosanoids	17-phenyl-18,19,20-trinor-prostaglandin D2	5.4253	5.5084	5.2946	5.4614	5.3166	5.4616	5.416	5.2954	5.3686	5.4771
184	Eicosanoids	Thromboxane B3	4.7457	1.0803	0.2828	3.6585	0.2934	5.8252	3.2524	0.2571	6.0249	4.7272

185	Terpenoids	Sandosapogenol	6.4278	6.3409	6.3441	6.4945	6.568	6.3404	6.4495	6.4287	6.314	6.3555
186	Terpenoids	Camelliagenin A	4.8929	4.9037	4.856	4.9208	4.9689	4.7575	4.9433	4.8953	4.9518	4.8227
187	Terpenoids	Theasapogenol E	3.6923	3.357	3.5674	3.6167	3.7716	3.611	3.6999	3.6335	3.6945	3.5576
188	Terpenoids	Glypallidifloric acid	4.3001	3.9116	3.7688	3.9638	3.8435	3.8434	3.8028	3.8352	3.7533	3.6767
189	Terpenoids	Erythrodiol 3-decanoate	5.951	6.0499	5.9393	6.1452	6.0843	5.9977	6.0105	6.0222	5.9474	5.9808
190	Terpenoids	Panaxatriol	5.7344	4.4199	4.4476	5.2166	4.6097	5.606	4.4378	4.3599	4.286	4.9303
191	Terpenoids	Theasapogenol A	5.4456	5.2352	5.4521	5.3659	5.5229	5.7043	5.5815	5.5569	5.1641	5.2691
192	Terpenoids	Phytolaccoside A	4.2903	4.2431	4.2526	4.326	4.3311	4.3682	4.3242	4.2479	4.2475	4.3067
193	Terpenoids	Lucidenic acid C	2.4221	2.4994	1.9437	2.7759	4.1525	3.6175	3.7564	4.2915	4.2722	4.6501
194	Terpenoids	Actinidic acid	1.6954	1.2605	1.2694	3.7821	3.6442	3.5265	2.1258	4.5542	2.886	4.1812
195	Terpenoids	Urs-13(18)-en-3beta-yl acetate	4.7342	4.561	4.1417	4.8145	6.0485	5.8022	5.0715	5.073	4.6023	4.7584
196	Terpenoids	3beta-Acetoxy-11alpha-methoxy-12-ursen-28-oic acid	3.2013	4.4442	4.1395	3.4879	3.552	3.9915	4.1695	3.1589	4.0888	3.7118
197	Terpenoid	Pitheduloside I	4.8906	4.5639	4.7933	4.8978	4.7572	5.0472	4.6295	4.4925	4.5966	4.8618

	s											
198	Terpenoids	(1beta,2alpha,3alpha)-1,2,3,24-Tetrahydroxy-12-oleanen-28-oic acid	3.2418	3.4362	3.2898	3.6196	3.4244	3.3948	3.3319	3.5896	3.4225	3.4504
199	Terpenoids	Cerebroside B	5.0492	4.2435	3.2897	5.4315	4.7875	4.4253	4.3891	4.376	3.4727	4.1555
200	Terpenoids	Ganoderic acid F	5.3146	5.1684	5.2134	5.4923	5.3777	4.9975	5.2481	5.3039	5.0476	5.4399
201	Terpenoids	Alpha-Amyrone	6.1381	5.6116	5.0312	6.299	5.839	5.6476	5.5546	5.3002	5.1366	5.6942
202	Terpenoids	Hydroxysintaxanthin 5,6-epoxide	5.1857	5.1924	5.1805	5.2819	5.2758	5.2046	5.2692	5.1798	5.2309	5.2481
203	Terpenoids	Lucyin A	3.2319	3.1506	3.0803	3.1449	3.2816	3.2658	3.2892	3.0971	3.1332	3.0217
204	Terpenoids	Longispinogenin	5.9435	5.755	5.9453	6.3019	6.1393	6.081	6.2022	6.1814	5.9133	5.8707
205	Terpenoids	Beta-Boswellic acid acetate	4.7706	4.3204	5.2938	4.7884	4.7435	4.7128	4.8514	4.9465	4.4793	4.5234
206	Terpenoids	Manglupenone	4.742	4.6841	4.6941	4.9798	4.7281	4.872	4.7678	4.6982	4.6926	4.6195
207	Terpenoids	Soyasapogenol A	4.5107	4.6604	4.3995	4.8267	5.2551	4.9521	4.7749	4.846	4.9501	4.777
208	Terpenoids	Heliantriol C	4.7015	4.6339	5.763	4.75	4.8598	4.8831	4.9409	4.5214	4.5473	4.5567
209	Terpenoids	3beta-Acetoxy-19alpha-hydroxy-12-ursene	6.229	5.8329	4.9302	5.7447	6.3774	6.1568	6.0709	6.2359	4.3517	5.5783

210	Terpenoids	16-Acetylpriverogenin A	5.9922	4.5421	3.7024	5.2807	6.3705	5.8047	6.0084	5.8472	3.7958	5.3726
211	Terpenoids	Gamma-Taraxastanonol	5.8845	5.2392	4.9202	6.0312	6.0213	5.635	5.4882	6.0639	4.9151	5.3425
212	Terpenoids	18-Dehydroursolic acid 3-arabinoside	6.4021	6.2461	6.5031	6.5223	6.42	6.4595	6.4144	6.375	6.2106	6.3209
213	Terpenoids	Ganoderiol B	5.0641	5.0584	5.019	5.4004	5.3218	5.243	5.1772	5.5035	4.9843	5.2387
214	Terpenoids	Sericoside	1.0181	1.41	2.0896	1.7147	2.0886	1.4363	2.0684	1.9314	2.2161	2.7231
215	Terpenoids	Ganoderol A	5.6876	5.7134	5.6181	5.6927	5.727	5.6011	5.6478	5.7108	5.6834	5.6738
216	Terpenoids	Camelliagenin B	5.2207	5.0225	4.9427	5.3956	5.3856	5.3289	5.3169	5.4966	4.8609	5.2827
217	Terpenoids	Lucidenic acid J	4.0219	2.9479	3.4308	3.1011	4.3765	3.5249	4.4169	3.3433	2.9522	4.4031
218	Terpenoids	Ganoderic acid G	5.5631	5.4111	5.4756	5.4488	5.5372	5.6313	5.422	5.2912	5.184	5.8008
219	Terpenoids	Glabric acid	7.6299	7.4359	7.6196	7.6335	8.2268	7.9631	8.0805	7.7788	7.6785	7.7966
220	Terpenoids	Hoduloside VII	5.8999	6.0208	5.9126	5.9406	5.9899	5.7552	5.9738	6.1402	5.7836	5.7838
221	Terpenoids	Hoduloside VI	3.6407	3.3159	4.5599	3.7189	3.1135	3.9651	3.4075	3.6754	3.4998	2.9458
222	Terpenoids	Mabioside D	4.7157	4.4025	4.4591	4.9862	4.6917	4.3826	4.5374	4.9575	4.1235	4.3157

223	Terpenoids	Hoduloside V	3.3722	4.1442	3.4016	4.0249	4.6638	3.1339	4.5863	4.5599	4.3393	4.5217
224	Terpenoids	Lucidenic acid F	5.7089	5.0336	4.8841	5.2876	5.7603	5.7467	6.1452	5.6164	4.8162	6.0344
225	Terpenoids	Ganoderic acid H	4.7501	4.8636	4.8301	4.8036	4.8154	4.6116	4.7729	4.8404	4.6341	4.8242
226	Terpenoids	Tragopogonsaponin M	4.5531	4.7057	4.5146	4.4572	4.5922	3.939	4.3952	4.7797	4.1508	4.1202
227	Terpenoids	Koryoginsenoside R1	3.521	4.3016	3.8272	3.1127	3.9705	3.1812	3.6709	3.9481	4.2992	3.8103
228	Terpenoids	Ganoderic acid beta	3.9869	3.3726	3.8349	4.0461	5.1868	5.368	4.7996	4.4868	4.0455	4.5421
229	Terpenoids	Corosin	5.8435	5.6413	5.8913	6.1555	6.5249	6.6287	6.2353	6.0057	5.8243	6.0756
230	Terpenoids	Ganoderenic acid C	3.9861	1.6679	2.5882	2.6815	5.4226	4.8493	3.9978	4.1171	3.6914	5.1871
231	Terpenoids	Medicagenic acid	6.0303	5.6148	5.7256	6.1178	6.5373	6.3184	5.976	6.0791	5.7037	5.9949
232	Terpenoids	Jujuboside B	6.6364	6.4655	6.4178	6.7447	6.4724	6.2417	6.3181	6.4884	6.3524	6.3795
233	Terpenoids	Tangeraxanthin	4.9504	4.5244	4.7938	4.5896	5.1396	5.1172	5.1377	4.9888	4.737	4.8855
234	Terpenoids	3-Oxo-12,18-ursadien-28-oic acid	5.8153	5.8412	5.8318	5.8577	5.7914	5.7212	5.8114	5.8354	5.8183	5.8154
235	Terpenoids	HEDERAGENIN	3.9065	4.4775	4.554	4.5143	4.5628	4.392	4.4869	4.2765	4.178	4.8596

236	Terpenoids	Soyasapogenol D	3.8305	3.6776	4.6822	5.7567	3.7896	5.0323	3.8833	3.9755	3.895	4.0729
237	Terpenoids	Ganoderiol I	6.1526	6.2402	6.1919	6.1688	6.2064	6.137	6.1834	6.2498	6.2478	6.1646
238	Terpenoids	Camellenodiol	4.6164	3.9755	4.3859	4.7911	5.2262	5.4582	5.0306	4.7728	4.5259	4.8471
239	Terpenoids	Ganodermanontriol	5.1772	4.3415	4.6692	5.4756	4.5935	4.4967	4.2372	4.4234	3.8676	5.2731
240	Terpenoids	2alpha-Hydroxypyracrenic acid	5.65	5.7731	5.7816	5.9549	6.0028	5.5659	5.599	5.8254	5.6894	5.5399
241	Terpenoids	Phytolaccinic acid	5.5932	5.6696	5.7771	5.7858	5.9178	5.5139	5.892	5.7745	5.7902	5.6319
242	Terpenoids	Ganoderenic acid D	3.8905	4.37	3.544	2.0925	5.0467	5.2384	3.4697	4.2302	4.7257	4.0326
243	Terpenoids	3-Benzoyloxy-6-oxo-12-ursen-28-oic acid	2.6551	1.7173	3.7443	4.5927	4.612	4.4803	3.193	4.6002	5.1089	5.5563
244	Terpenoids	Pubesenolide	4.4041	4.1383	3.9379	0.5419	5.4816	5.678	4.5007	4.9551	4.9579	4.9456
245	Terpenoids	Phytolaccoside D	4.0326	3.4661	3.5095	4.2658	4.6969	3.9084	5.8571	4.6094	3.4698	4.6762
246	Terpenoids	Notoginsenoside T2	3.2456	3.1967	4.3857	3.3995	3.2621	5.7011	4.6677	3.1847	3.2004	3.2389
247	Terpenoids	Ganoderic acid A	4.0717	4.0228	4.0662	4.0918	4.0882	4.1209	4.0745	4.0107	4.0264	4.065
248	Terpenoids	Physalin A	4.097	3.5528	3.1128	3.1461	3.937	3.5464	4.0006	5.3466	3.0731	3.1853

249	Terpenoids	3-Hydroxy-10'-apo-b,y-carotenal	3.3436	3.3915	3.1947	3.5017	3.4508	3.5832	3.4278	3.6284	3.4396	3.598
250	Terpenoids	(9-cis,9'-cis)-7,7',8,8'-Tetrahydro-psi,psi-carotene	6.3226	6.515	6.5133	6.4763	6.4348	6.363	6.4607	6.9744	6.3294	6.3016
251	Terpenoids	7,7',8,8'-Tetrahydro-beta,beta-carotene	5.8721	5.7102	5.8333	6.0022	5.8363	5.8493	5.9597	6.5156	5.6996	5.7347
252	Terpenoids	1,2-Epoxy-1,2,7,7',8,8',11',12'-octahydro-psi,psi-carotene	2.8607	2.8135	2.823	2.8964	2.9015	6.1084	3.0301	3.103	2.8179	2.8771
253	Terpenoids	9alpha-(3-Methylbutanoyloxy)-4S-hydroxy-10(14)-oplopen-3-one	5.9228	5.5889	5.5875	6.2495	5.7806	5.9727	5.752	5.2273	5.3378	6.126
254	Terpenoids	Auberganol	4.3767	4.1507	4.7273	4.5396	4.5242	4.6642	4.4835	4.1598	4.2148	4.453
255	Terpenoids	Ganoderic acid S	3.5292	3.8763	3.0329	4.1903	4.7172	4.7382	3.4194	3.122	3.9842	4.7494
256	Terpenoids	Tanacetol A	3.5658	3.2503	3.3579	4.2176	3.5562	3.7719	3.6578	4.0158	3.6887	3.6393
257	Terpenoids	Capsidiol	5.1805	5.0551	5.0603	5.1439	5.416	5.4595	5.1936	5.0694	4.9852	5.1852
258	Terpenoids	Tocopheronic acid	4.9911	5.0604	4.874	4.9721	4.9641	5.0754	5.096	4.7623	4.9888	5.0668
259	Terpenoids	Zedoarol	6.785	6.9026	6.5282	6.4433	6.8812	6.8405	6.7504	6.7991	6.9758	6.9624
260	Terpenoid	(R)-2,7(14),9-Bisabolat	4.2559	3.8196	4.6815	5.1059	4.2547	4.7032	4.5204	3.9638	4.0088	4.2843

	s	rien-11-ol										
261	Terpenoids	Melledonal A	5.8097	6.3403	5.6279	5.5052	5.9083	5.9914	6.2375	5.8752	6.1441	6.0247
262	Terpenoids	(3beta,6beta)-Furanoeremophilane-3,6-diol	4.6765	4.7367	4.6275	4.6729	4.964	5.0675	4.8002	4.5455	4.6663	4.7622
263	Terpenoids	Lepidiumterpenyl ester	4.4002	4.6325	4.2393	5.1224	4.728	4.7796	5.0063	4.7944	4.7158	4.714
264	Terpenoids	(+)-4,11-Eudesmadien-3-one	3.1186	3.2973	3.0968	3.2393	3.0795	3.1343	3.1032	3.0588	3.0678	3.0959
265	Terpenoids	2,6,10,15-tetramethylheptadecane	4.1101	4.0629	4.0724	4.1458	4.1509	5.5288	4.144	4.0677	4.0673	4.1265
266	Terpenoids	Ar-Artemisene	5.1207	3.2221	2.7706	3.5408	4.1337	3.2919	3.5356	3.0619	2.8295	3.7946
267	Terpenoids	4,5-Dihydrovomifoliol	4.7379	5.2336	5.1133	5.0085	4.9829	5.0902	5.1446	4.6988	4.9871	5.1708
268	Terpenoids	13-Nor-6-eremophilene-8,11-dione	2.8733	2.8262	2.8356	2.909	3.1208	3.2257	2.9072	2.8309	2.8305	3.0016
269	Terpenoids	Deterrol stearate	5.1262	3.9691	3.8766	5.2513	4.9195	5.4704	5.1747	4.5268	3.5713	4.4342
270	Terpenoids	Tanacetol B	6.2271	6.2829	6.1981	6.2897	6.2901	6.2226	6.2675	6.2955	6.3001	6.2631
271	Terpenoids	Germacrenone	5.6096	5.7656	5.8746	5.6166	5.7348	5.6715	5.7274	5.6393	5.6682	5.7075
272	Terpenoids	Armillaritin	6.2796	6.5223	6.4816	6.091	6.2039	6.0178	6.0641	6.0346	6.3568	6.0579
273	Terpenoid	(E)-Calamene	2.9671	3.2433	3.0809	3.0286	3.4097	4.9929	3.0616	2.9172	2.9393	4.0449

	s											
274	Terpenoids	7-Hydroxycostol	4.3637	4.3307	4.3828	4.4764	4.4982	4.6224	4.4877	4.1482	4.0797	4.2916
275	Terpenoids	Alpha-curcumene	5.0233	4.8766	4.9437	5.0837	5.1636	5.0338	4.9931	4.9825	4.8658	5.0115
276	Terpenoids	Lucidenic acid A	4.229	3.3751	3.6185	3.7778	3.9852	3.9801	3.7945	3.3904	2.9288	3.4608
277	Terpenoids	Pyrocurzerenone	5.0822	4.9672	4.9703	5.3501	5.0363	5.0887	4.9462	4.9831	4.9705	5.0648
278	Terpenoids	3-Hydroxy-beta-ionone	4.6139	4.6097	4.547	4.6307	4.6472	4.6715	4.5822	4.684	4.583	4.5663
279	Terpenoids	Isorhamnetin 3-glucoside	4.3948	4.3187	4.6035	4.2652	4.4422	4.1795	4.046	4.1299	4.5488	4.4559
280	Terpenoids	3,7,8,15-Scirpenetetrol	4.0328	4.412	3.7292	3.5351	4.1412	4.2572	4.3193	4.1365	4.5487	4.6046
281	Terpenoids	Procurcumadiol	1.4411	1.3956	1.4047	2.4737	1.4805	1.5163	1.4738	1.4002	1.3998	1.4569
282	Terpenoids	Beta-Ionone	3.985	3.9878	3.9928	4.105	4.0596	3.996	4.023	3.8987	3.8569	3.9693
283	Terpenoids	Heterocodeine	5.4259	5.8194	5.4576	4.8581	5.445	5.5956	5.5686	5.8064	5.6521	5.5266
284	Terpenoids	Tricyclohumuladiol	4.0514	2.8569	4.553	5.4083	4.43	4.4698	4.4467	2.7682	2.7678	2.9729
285	Terpenoids	Artelinic acid	2.3484	3.4478	3.8492	4.0014	3.7643	4.3635	3.7821	3.795	2.5684	2.5029
286	Terpenoid	Glandulone B	4.2871	3.9436	4.2444	4.3276	4.1595	4.2783	4.0677	4.2456	4.1298	4.2903

	s											
287	Terpenoids	Sugeonol	4.5616	3.3785	3.328	4.8877	4.5967	4.1381	4.4081	4.1864	2.6483	2.8711
288	Terpenoids	7alpha,8alpha-Dihydroxycalonectrin	4.3591	4.3649	4.3139	4.3088	4.315	4.5404	4.3703	4.0582	4.2797	4.3875
289	Terpenoids	Dehydrovomifoliol	4.4057	4.3604	4.358	4.3478	4.4462	4.4068	4.3506	4.3425	4.3225	4.3902
290	Terpenoids	Armillyl orsellinate	5.9531	6.0309	6.0043	6.238	6.0646	6.0693	6.1503	5.9085	5.9726	5.8402
291	Terpenoids	Armillarivin	6.0559	5.8173	5.7152	6.1541	5.7815	5.9793	5.873	5.6903	5.7964	5.8451
292	Terpenoids	8,12-Epoxy-4(15),7,11-eudesmatrien-1-one	5.162	4.3151	4.1085	4.6216	5.0659	5.2002	4.5902	4.0834	4.4507	4.4428
293	Terpenoids	T2 Triol	4.6962	4.2815	4.2447	4.4653	4.329	3.9924	4.558	4.3286	3.8216	4.2168
294	Terpenoids	Deoxynivalenol 3-glucoside	4.4999	4.5025	4.8039	4.759	4.4721	4.3469	4.5842	4.4523	4.9105	4.8087
295	Terpenoids	Nivalenol	4.5032	4.5064	4.4926	3.9817	4.6506	4.574	4.5001	4.5496	4.6037	4.4547
296	Terpenoids	Melleolide F	4.7214	4.5502	4.8065	5.3786	4.823	4.8665	4.8191	4.5758	4.4175	4.5979
297	Terpenoids	(3S,5R,6R,7E)-3,5,6-Trihydroxy-7-megastigmen-9-one	3.9714	3.9108	4.2651	4.4412	4.3581	4.391	4.4023	4.0033	2.7929	4.0562
298	Terpenoids	Methyl (3b,11x)-3-Hydroxy-8-ox	5.4681	5.579	5.3076	5.4645	5.4417	5.3868	5.4958	5.5756	5.5397	5.4988

		o-6-eremophilen-12-oate										
299	Terpenoids	(1(10)E,4a,5E)-1(10),5-Germacradiene-12-acetoxy-4,11-diol	3.2657	3.741	3.5714	4.2796	3.6046	3.4404	3.4974	3.7382	3.6726	3.7703
300	Terpenoids	4-Hydroxy-3-methoxy-2,10-bisaboladien-9-one	5.4546	4.8971	5.1839	5.2612	5.56	6.1435	5.8764	4.9366	4.7384	5.0876
301	Terpenoids	Armillatin	4.1702	4.1213	4.1647	6.0662	4.2023	4.2194	4.173	4.1092	4.125	4.1635
302	Terpenoids	Melleolide	5.9283	5.7986	5.782	5.959	5.7909	5.9905	5.8469	5.8048	5.782	5.9486
303	Terpenoids	(17alpha,23S)-17,23-Epoxy-29-hydroxy-27-norlanosta-1,8-diene-3,15,24-trione	4.8834	3.9321	4.0497	4.6594	3.6243	4.5571	3.6579	2.299	1.4753	3.1578
304	Terpenoids	Gamma-Eudesmol rhamnoside	3.2489	3.2	3.2434	3.3661	3.2654	3.2981	3.2517	3.1879	3.2037	3.2422
305	Terpenoids	4-(2,6,6-Trimethyl-1-cyclohexenyl)-2-butanol	4.1946	3.693	3.9306	4.8088	4.7032	4.1525	4.3295	3.5009	3.6707	3.5147
306	Terpenoids	(3S,4S,6R,7S)-1,10-Bisaboladiene-3,4-diol	5.1039	5.1077	5.1024	5.3565	5.3065	4.6599	5.6373	4.8989	5.0754	4.9772
307	Terpenoids	Dehydro-beta-Ionone	3.8316	2.4595	3.7636	3.2014	5.2962	4.473	4.5392	4.8743	2.638	3.3042
308	Terpenoids	Ipomeatetrahydrofuran	6.0971	6.0752	5.9584	6.0048	6.2295	6.2594	6.1742	5.8671	5.9535	5.8447
309	Terpenoids	Bisacurone epoxide	4.0533	4.028	4.1648	4.2218	4.3815	4.2559	4.3262	3.8102	3.2453	4.048

310	Terpenoids	Pisumic acid	4.2358	4.1362	4.3649	4.4896	4.2212	4.2693	4.3108	4.196	3.9753	4.0498
311	Terpenoids	Sterebin A	5.3007	5.3237	5.5758	5.5256	5.3571	5.8344	5.5239	5.3629	5.3898	5.1498
312	Terpenoids	7(14)-Bisabolene-2,3,10,11-tetrol	4.5374	4.4041	5.0089	4.5989	4.9703	4.9924	4.8388	4.1397	3.5916	4.3173
313	Terpenoids	Piperoic acid	5.4944	5.5416	5.8021	5.7185	5.4658	5.4023	5.6675	5.6043	5.5945	5.5781
314	Terpenoids	Eremopetasidione	6.4375	6.0492	6.2224	6.5419	6.094	6.1867	6.0803	6.078	5.7936	6.0197
315	Terpenoids	6Z-8-Hydroxygeraniol 8-O-glucoside	5.2056	5.2322	4.6157	4.5311	4.8465	4.9161	5.0776	4.6726	4.9435	4.8909
316	Terpenoids	(3alphaOH,20S,24S)-3,19:20,24-Diepoxydammarane-3,25-diol	5.1698	5.2948	5.2149	5.4714	5.2038	5.2666	5.3282	5.1275	5.1711	5.0159
317	Terpenoids	N-[(4E,8Z)-1,3-dihydroxyoctadeca-4,8-dien-2-yl]hexadecanamide 1-glucoside	5.0915	5.402	5.2559	5.2864	5.1117	5.1153	5.1235	5.3758	5.2076	5.0226
318	Terpenoids	Acuminoside	5.1074	5.0112	4.9211	5.617	5.2666	4.929	4.8355	4.8988	4.9786	4.9939
319	Terpenoids	6-Hydroxy-2-bornanone glucoside	4.8972	5.5333	4.8531	5.0255	5.0016	5.1028	5.4967	4.56	4.9456	4.9561
320	Terpenoids	(1S,2R,4R)-p-Menth-8-ene-2,10-diol 2-glucoside	4.485	4.1467	4.4958	3.89	4.0686	4.9501	4.3383	4.1406	4.3384	3.9413
321	Terpenoid	3-Hydroxy-beta-ionol	4.5413	4.3064	4.6467	4.6136	4.4588	4.179	4.7063	4.6402	4.6129	4.646

	s	3-[glucosyl-(1->6)-glucoside]									
322	Terpenoids	Oleoside 11-methyl ester	3.4202	3.2594	4.1391	3.22	3.5729	3.8031	3.9721	3.7305	3.7859	3.8904
323	Terpenoids	(1R,2R,4S,6R)-2,6-Fenchanediol 2-O-b-D-glucoside	3.5375	3.4841	3.7458	3.5046	3.2886	3.4075	3.554	3.2793	3.3974	3.6492
324	Terpenoids	10-Hydroxy-8-nor-2-fenchanone glucoside	4.2837	4.2614	4.3835	5.4028	4.6049	4.2812	4.9094	4.4269	4.3382	4.5431
325	Terpenoids	Agnuside	4.1822	4.135	4.1445	4.2179	4.223	4.2601	4.2161	4.1398	4.1394	4.1986
326	Terpenoids	Icariside B8	4.93	4.0148	4.1724	4.0175	3.871	5.3684	4.294	4.1019	5.5233	4.6549
327	Terpenoids	Goshonoside F3	5.4009	5.3537	5.3632	5.4365	5.4417	5.4787	5.4348	5.3584	5.358	5.4173
328	Terpenoids	(4S,6R)-p-Mentha-1,8-diene-6,7-diol 7-glucoside	3.642	2.7983	3.6787	4.2128	3.4058	3.7557	3.2491	2.6879	2.8504	2.5871
329	Terpenoids	Ichangic acid 17-beta-D-glucopyranoside	4.174	3.2385	3.3156	3.507	3.3123	3.2874	3.539	3.3674	3.7146	3.226
330	Terpenoids	Pitheduloside A	5.109	4.8448	4.7335	5.2167	5.0297	4.6547	4.8232	4.8891	4.7583	4.8677
331	Terpenoids	Soyasaponin IV	4.8227	4.8371	4.7506	4.8254	4.8723	4.7645	4.7832	4.7629	4.8506	4.7935
332	Terpenoid	Perilloside C	5.2654	5.3191	5.1939	5.3686	5.3039	5.2322	5.3388	5.3758	5.3735	5.279

	s											
333	Terpenoids	Saponin H	4.2761	4.3794	4.238	4.3422	4.1864	4.0628	4.2869	4.2968	4.3194	4.3975
334	Terpenoids	Aucubin	3.2699	4.3275	3.2567	2.8568	3.6819	4.0356	4.2627	3.3762	4.2537	4.0393
335	Terpenoids	Cinncassiol A 19-glucoside	4.8871	5.355	4.9704	4.9191	4.9452	5.0389	5.2734	4.7376	5.1359	5.1141
336	Terpenoids	Citronellyl beta-sophoroside	1.9635	1.2988	1.8917	1.3779	2.8311	2.9826	1.9991	1.3033	1.8983	3.2622
337	Terpenoids	4-Hydroxyretinoic acid glucuronide	4.4933	5.2072	4.4648	4.104	4.5836	4.8957	5.1794	4.6664	5.0525	4.8299
338	Terpenoids	Cinnamoside	0.3308	0.3875	0.6338	0.3504	0.659	1.0081	1.1354	0.4833	1.2475	1.4487
339	Terpenoids	Camelliasaponin A1	2.023	1.3414	1.3504	2.0581	1.4258	1.4615	1.4191	1.3459	1.3455	1.4023
340	Terpenoids	Cofaryloside	0.435	1.5524	2.1455	1.5889	0.8965	0.3143	1.0365	1.4187	0.8269	1.4572
341	Terpenoids	Lippioside II	4.4201	4.1891	5.1819	4.5951	3.844	4.4219	4.4784	4.8333	5.2295	4.3346
342	Terpenoids	Oleoside dimethyl ester	1.8136	0.0008	1.7948	2.969	0.9263	1.0783	0.1256	1.3297	0.7811	0.0053
343	Terpenoids	Gibberellin A1 glucosyl ester	4.672	5.108	4.851	4.7279	4.8327	4.6559	4.8389	5.2441	5.288	5.4559
344	Terpenoids	Indole-3-acetic-acid-O-glucuronide	3.4414	3.7597	4.2972	3.7352	4.0344	3.6865	3.9723	4.1593	4.4384	4.2441
345	Terpenoid	Gibberellin A20	5.0533	5.1223	5.0443	4.5043	5.149	4.8187	5.1277	5.0199	5.2335	5.3697

	s	13-glucoside										
346	Terpenoids	Pisumionoside	4.4849	4.0764	4.3505	3.7763	3.6671	4.7855	3.5896	3.9081	4.8837	4.9102
347	Terpenoids	(1S,2R,4R,8S)-p-Menthane-2,8,9-triol 9-glucoside	4.3345	4.6311	4.6694	4.6152	4.6446	4.2729	4.5184	4.7624	4.5453	4.5378
348	Terpenoids	Ichangin 4-glucoside	6.0449	6.0325	6.1478	6.1629	6.2401	5.9208	6.0867	6.2748	5.9357	5.996
349	Terpenoids	(4R,5S,7R,11x)-11,12-Dihydroxy-1(10)-spirovetiven-2-one 12-glucoside	3.8508	3.2749	4.4324	4.5009	3.8119	5.1322	3.8363	3.6221	3.1926	3.8113
350	Terpenoids	Maslinic acid 3-O-b-D-glucoside	1.9078	3.8652	3.9314	4.2377	3.2983	4.1031	4.1401	3.1346	3.7199	3.6782
351	Terpenoids	2a-Hydroxygypsogenin 3-O-b-D-glucoside	4.0969	4.3512	3.5795	5.5511	4.6831	4.9349	4.8048	4.7633	3.5646	4.3018
352	Terpenoids	(-)-trans-Carveol glucoside	3.2922	3.5256	4.2873	2.4645	3.5546	3.8006	3.0301	4.0161	3.9854	4.3791
353	Terpenoids	(1S,2R,4R,8S)-p-Menthane-2,8,9-triol 2-glucoside	4.1054	4.2063	4.1417	4.5822	4.1475	4.486	4.4107	4.3142	4.3703	3.6789
354	Terpenoids	(1S,3R,4R)-8,10-Dihydroxyfenchone 10-O-b-D-glucoside	3.6633	3.9514	4.179	4.0563	4.1685	3.9385	4.1033	4.0094	3.9711	3.7983
355	Terpenoids	Monotropein	5.3555	5.5952	5.7817	5.4427	5.3254	5.2689	5.4678	5.5518	5.4008	5.3845
356	Terpenoid	Nepetaside	3.2436	0.5852	3.6421	3.6261	3.1344	2.1668	1.9266	3.0494	0.0054	2.5337

	s											
357	Terpenoids	Geniposidic acid	3.5527	4.1063	4.0147	3.6571	3.6945	3.6311	4.0485	3.8156	3.9234	3.7212
358	Terpenoids	Ent-6R,16bOH,17-Trihydroxy-7-oxo-6,7-seco-19,6-kauranolide 6-O-glucoside	5.2573	5.3732	5.4839	5.6369	5.3142	5.5986	5.424	5.6235	5.3876	5.1967
359	Terpenoids	Tsangane L 3-glucoside	2.7703	2.7214	2.7648	2.7903	3.4688	2.8636	2.8023	2.7094	3.7559	2.8657
360	Terpenoids	Kudzusaponin SA1	3.8902	3.4551	3.561	3.8629	3.613	3.5532	4.3418	3.6976	3.5056	3.8431
361	Terpenoids	20-Deoxynarasin	5.1083	4.8155	4.726	4.9934	4.9429	4.5486	4.6462	5.0267	4.7093	4.7304
362	Terpenoids	Lucyoside N	3.1013	3.0524	4.1467	4.0873	3.1178	5.0106	3.1042	3.0404	3.0561	3.0946
363	Terpenoids	Majonoside R2	3.3574	3.3085	4.5867	3.4925	3.3739	4.5944	3.3603	3.2965	3.3122	3.3507
364	Terpenoids	Licoricesaponin D3	5.7281	5.6792	5.7226	5.7482	5.7446	5.7773	5.731	5.6671	5.6829	5.7214
365	Terpenoids	Cynarasaponin H	5.7684	5.132	4.852	5.3457	5.82	5.8585	6.1186	5.6964	4.8439	6.0681
366	Terpenoids	Neryl arabinofuranosyl-glucoside	3.6282	3.4757	3.9771	4.0503	4.3899	4.5029	3.6626	4.3491	4.4825	4.7675
367	Terpenoids	28-Glucosylarjunolate 3-[rhamnosyl-(1->3)-glu	5.4073	4.8914	4.725	4.8755	5.5391	5.3938	5.7201	5.3566	4.395	5.5727

		curonide]									
368	Terpenoids	Menthol-glucoronide	4.7916	4.6257	4.7281	5.0361	5.1308	4.9792	5.0872	4.3687	4.2531	4.7299
369	Terpenoids	Medicoside L	4.7583	4.9306	4.9935	4.8213	5.4327	4.8927	5.1455	4.9657	4.8801	5.0344
370	Terpenoids	(1R*,2R*,4R*,8S*)-p-Menthane-1,2,8,9-tetrol 9-glucoside	4.5932	1.8925	4.4502	0.2311	4.1965	0.571	4.1066	1.9869	1.0403	3.7909
371	Terpenoids	Cinncassiol D2 glucoside	5.8802	5.459	5.9224	6.1347	5.6395	5.6981	5.5708	5.721	5.8666	5.6584
372	Terpenoids	Assamsaponin B	4.9415	4.8927	4.9361	4.9617	4.9581	4.9908	4.9444	4.8806	4.8963	4.9349
373	Terpenoids	Sonchuionoside C	5.2437	5.4166	5.5455	5.7773	5.5034	5.3971	5.4233	5.5124	5.3612	5.3188
374	Terpenoids	Kiwiionoside	5.8876	6.0365	6.2659	6.1283	5.9921	5.8378	5.9444	5.9121	5.923	5.8662
375	Terpenoids	Trans-p-Menth-2-ene-1,4-diol	5.0419	5.0382	5.0183	5.0048	5.0826	5.1937	5.0409	5.035	5.0472	5.0496
376	Terpenoids	Isobornyl 2-methylbutyrate	4.7921	4.7075	4.7179	4.7377	4.9967	4.9301	4.8371	4.7296	4.521	4.7154
377	Terpenoids	Limonene aldehyde	4.6016	5.3542	5.0928	5.0729	4.4685	5.3352	4.7009	4.8425	4.4484	4.744
378	Terpenoids	3-Caren-5-one	5.6247	5.6121	5.6297	5.6432	5.7044	5.6888	5.6566	5.6013	5.5973	5.6282
379	Terpenoids	4-(4-Methyl-3-pentenyl)-3-cyclohexene-1-carbox	4.1267	4.1818	4.1249	4.1532	4.1569	4.1494	4.238	4.2358	4.0438	3.9789

		aldehyde										
380	Terpenoids	Alpha-Terpinene	6.262	6.2921	6.2276	6.371	6.3045	6.3348	6.3216	6.2806	6.2188	6.291
381	Terpenoids	(S)-(-)-Perillyl alcohol	4.2379	4.303	4.3568	4.3541	4.352	4.3492	4.3084	4.3243	4.2999	4.2508
382	Terpenoids	Vulgarole	5.3859	5.386	5.3099	5.4495	5.4472	5.3571	5.3578	5.3026	5.3332	5.3197
383	Terpenoids	Blumealactone A	3.5841	4.7024	4.841	3.8	3.9849	3.9767	4.3581	3.6471	4.6719	3.975
384	Terpenoids	Dihydrocarveol acetate	3.7198	3.8356	3.8407	3.6362	3.9506	3.8532	3.9021	3.4999	3.7631	3.7508
385	Terpenoids	(4S,8R)-8,9-Dihydroxy-p-menth-1(6)-en-2-one	5.532	5.5505	5.5066	5.573	5.5928	5.542	5.6066	5.5689	5.5924	5.5337
386	Terpenoids	1-Acetyl-2-methylcyclopentene	5.252	4.1658	4.2987	3.8178	4.7923	3.9979	4.4621	4.7771	4.4592	4.51
387	Terpenoids	(x)-p-Menth-1-en-4-yl 5-isopropyl-2-methylphenyl ether	4.5808	4.8637	4.8335	4.4912	4.2468	4.7046	4.5711	4.409	4.6344	4.6413
388	Terpenoids	3-(L-Menthoxy)-2-methylpropane-1,2-diol	3.0901	3.5183	2.9219	3.0813	3.0253	3.1425	3.3617	2.9172	3.005	3.3278
389	Terpenoids	3-(4-Isopropylphenyl)propanal	2.9179	3.0666	3.1648	3.1267	3.4701	3.0773	2.9459	2.8538	3.1231	3.4459
390	Terpenoids	Alpha-Campholene acetate	4.3403	4.4799	4.5375	4.5191	4.6512	4.3683	4.5087	4.4025	4.3037	4.358
391	Terpenoids	Citronellyl anthranilate	4.8801	4.8849	4.9476	4.9674	5.059	4.9973	5.008	5.1038	5.014	4.9004

392	Terpenoids	Alpha-Terpineol propanoate	7.0202	6.9055	6.9228	6.9932	7.0013	6.9773	6.9932	7.0107	6.9379	7.0743
393	Terpenoids	4-Isopropyl-3-cyclohexene-1-carboxylic acid	5.3085	5.3092	5.3351	5.3312	5.3775	5.4308	5.3215	5.324	5.2883	5.3357
394	Terpenoids	L-Menthyl (R,S)-3-hydroxybutyrate	3.1175	2.9575	3.1154	3.7262	3.8342	4.017	3.5948	2.9961	2.8122	3.8992
395	Terpenoids	Cuminaldehyde	4.6931	4.6399	4.6405	4.8617	4.6901	4.7295	4.6407	4.6401	4.6113	4.6352
396	Terpenoids	Pseudoionone	4.1791	4.2704	4.166	4.1522	4.1929	4.1964	4.1845	4.2732	4.2651	4.2449
397	Terpenoids	Linalyl phenylacetate	4.6222	4.3502	4.2584	4.6371	4.534	4.529	4.8191	5.1315	4.2973	4.2474
398	Terpenoids	(1R,2R,4R)-1,8-Epoxy-p-menthane-2,4-diol	4.8234	4.7812	4.8382	5.0248	4.7841	4.8757	4.8479	4.7862	4.5255	4.6779
399	Terpenoids	(1beta,2beta,5beta)-p-Menth-3-ene-1,2,5-triol	3.4469	3.8076	4.2877	4.2639	3.9118	4.308	4.604	3.9369	3.2734	4.1921
400	Terpenoids	Withangulatin A	4.9741	5.0444	5.5689	4.8823	4.5134	4.8325	5.1226	4.9402	4.5352	4.8935
401	Terpenoids	2-Hydroxy-p-mentha-1,8-dien-6-one	4.2905	4.2035	4.4395	4.479	4.2085	4.301	4.0129	4.1334	3.7151	3.7557
402	Terpenoids	2,2,4,4-Tetramethyl-6-(1-oxobutyl)-1,3,5-cyclohexanetrione	4.9351	4.944	5.0686	5.1436	5.216	5.1615	5.146	5.0061	4.7892	4.8142
403	Terpenoids	Gamma-Terpinene	6.4143	6.4418	6.3964	6.4999	6.4546	6.4758	6.457	6.418	6.3677	6.4307
404	Terpenoid	Geraniol	5.7759	5.7798	5.7542	6.0019	5.9242	5.9663	6.032	5.9497	5.8945	5.8585

	s											
405	Terpenoids	Monomenthyl succinate	4.9208	4.9344	5.2411	5.9199	5.1394	5.7303	5.0149	4.8528	4.7691	5.0139
406	Terpenoids	2-Hydroxypropyl 2-isopropyl-5-methylcyclohexyl carbonate	4.7585	4.7077	4.9599	4.8303	5.0086	5.0641	4.8427	4.8035	4.9727	4.9461
407	Terpenoids	Genipin	4.8106	4.9748	4.7322	4.7986	4.8693	4.6205	4.7675	4.8869	4.9862	5.1965
408	Terpenoids	(1S,2S,4R,8R)-p-Menthane-1,2,8,9-tetrol	4.9677	4.9866	4.5904	4.6171	5.3202	4.7938	4.8685	4.6939	4.9999	5.1609
409	Terpenoids	(-)-Pinocarvone	3.979	3.8237	4.1406	3.9693	4.6378	4.1857	4.1285	3.807	3.3624	4.1635
410	Terpenoids	Helinorbisabone	5.4257	5.2602	5.5803	5.6998	5.368	5.05	5.4487	5.3281	5.3201	5.4769
411	Terpenoids	(S)-Oleuropeic acid	3.9333	3.9629	3.9656	3.7976	4.0548	4.2221	4.0163	4.3136	4.2146	4.4304
412	Terpenoids	3-[[5-Methyl-2-(1-methylethyl)cyclohexyl]oxy]-1,2-propanediol	2.9415	3.6698	4.5986	4.2953	3.9875	4.9015	4.2882	3.994	2.7108	4.0186
413	Terpenoids	Veranisatin C	4.9131	4.8386	4.7822	4.6785	4.942	4.7913	4.7374	4.9223	4.8745	4.7767
414	Terpenoids	Armexifolin	5.3961	4.8802	5.1593	5.3871	5.2501	5.52	5.1958	4.8596	5.1757	5.2278
415	Terpenoids	Dehydrocyanaropicrin	4.1449	4.0417	4.126	4.4395	4.2096	3.7093	4.0445	4.2862	3.6304	3.8108
416	Terpenoid	Cynaratriol	5.0591	4.9019	4.9356	4.8191	5.0049	5.1609	5.0256	5.2768	5.1705	5.2137

	s											
417	Terpenoids	3b-Hydroxy-6b-(3-chloro-2-hydroxy-2-methylbutanoyloxy)-7(11)-eremophilen-12,8b-olide	5.2706	4.482	4.5278	5.2961	4.5203	3.7577	4.6709	4.3565	4.1197	4.5702
418	Terpenoids	Cynaroside A	5.2671	5.2316	5.1314	4.8437	5.2971	5.5018	5.1509	5.213	5.2669	4.9149
419	Terpenoids	8beta-Angeloyloxy-15-hydroxy-1alpha,10R-dimethoxy-3-oxo-11(13)-germacren-12,6alpha-olide	4.9418	5.2139	4.8983	5.4366	5.1354	5.2414	5.2586	4.9571	4.9854	4.7988
420	Terpenoids	3,8-Dihydroxy-6-methoxy-7(11)-eremophilen-12,8-olide	5.72	5.7134	5.6635	5.7491	5.758	5.7154	5.7757	5.735	5.7517	5.7015
421	Terpenoids	Arlatin	4.7025	4.7947	4.7089	4.7917	4.8122	4.8171	4.7908	4.7561	4.7911	4.7987
422	Terpenoids	(6beta,8betaOH)-6,8-Dihydroxy-7(11)-eremophilen-12,8-olide	5.302	5.3604	5.2481	5.3765	5.3808	5.3038	5.329	5.3915	5.3606	5.3542
423	Terpenoids	1alpha-O-Methylquassin	5.5126	5.1153	5.2228	5.9556	5.2584	5.273	5.0645	5.0593	5.0238	5.241
424	Terpenoids	Epinepetalactone	4.5598	4.5981	4.6247	4.6256	4.6327	4.6899	4.5934	4.5397	4.506	4.521
425	Terpenoids	Tavulin	4.9058	4.4703	4.6819	5.0754	4.598	4.7011	4.544	4.5788	4.1962	4.4749
426	Terpenoid	Taraxinic acid glucosyl	3.7311	4.5671	3.462	3.8976	3.612	3.8524	4.4203	3.4216	3.8261	3.7558

	s	ester										
427	Terpenoids	3b,8b-Dihydroxy-6b-(3-chloro-2-hydroxy-2-methylbutanoyloxy)-7(11)-eremophilen-12,8-olide	4.1664	2.9573	4.849	4.5882	4.4912	4.6945	3.8277	3.0284	2.9616	4.2045
428	Terpenoids	3,14-Dihydroxy-11,13-dihydrocostunolide	4.3445	4.2189	4.2542	3.9136	5.1222	5.3908	4.7682	4.4158	4.5235	4.9129
429	Terpenoids	Oryzalide A	4.2744	4.2272	4.2367	4.3101	4.3152	4.3522	4.3083	4.232	4.2316	4.2908
430	Terpenoids	Rosmaricine	4.6314	4.6429	3.4301	3.6876	4.9545	4.4308	4.4029	4.7167	4.6835	4.9781
431	Terpenoids	(1E,4Z,6a,8b,10a)-8-Angeloyloxy-10,15-dihydroxy-3-oxo-1,4,11(13)-germacratrien-12,6-olide	5.3994	5.6256	5.2033	5.1642	5.4008	5.604	5.6408	5.3104	5.7513	5.482
432	Terpenoids	(6beta,7alpha,12beta,13beta)-7-Hydroxy-11,16-dioxo-8,14-apianadien-22,6-olide	5.5035	5.7658	5.4483	5.5829	5.6134	5.6493	5.6563	5.4827	5.6548	5.7083
433	Terpenoids	4,5-Dihydroniveusin A	5.7109	5.6591	5.7583	5.7396	5.4181	5.6774	5.5277	5.4074	5.719	5.5554
434	Terpenoids	Hydroxyisonobilin	3.5248	3.5404	3.8075	3.7864	3.5987	4.0023	3.7578	3.3638	3.3827	3.0665
435	Terpenoids	Cinncassiol A	5.0582	5.012	5.1555	5.2673	5.0869	5.1783	4.965	5.2551	5.0694	4.9063
436	Terpenoid	3b-Hydroxy-6b-methoxy-7	3.7968	3.7691	4.9184	4.5874	2.8356	5.0876	3.3349	4.33	3.322	4.0634

	s	(11)-eremophilen-12,8a-olide										
437	Terpenoids	Artemin	4.2656	3.6136	3.6758	3.7767	4.8493	4.933	4.4652	3.6538	3.4927	4.1553
438	Terpenoids	Annuolide E	4.8471	4.8487	4.8686	4.8823	4.8164	4.7236	4.8516	4.8849	4.8558	4.8751
439	Terpenoids	Lactaronecatorin A	5.6013	4.9405	5.3995	5.0524	5.572	5.7167	5.3248	5.2006	4.9903	5.5278
440	Terpenoids	Crispolide	3.8817	3.7186	4.7968	3.8184	4.5231	4.403	4.0996	3.8749	3.7047	4.195
441	Terpenoids	Quassimarin	5.5674	5.8969	5.4029	5.372	5.6945	5.7552	5.7055	5.5063	5.6136	5.6708
442	Terpenoids	Gibberellin A88	3.0417	2.6975	2.5987	3.0994	2.7245	2.6579	2.8942	2.9679	2.9865	2.9963
443	Terpenoids	Bakkenolide D	2.3232	3.1557	3.3421	3.2242	2.7924	2.6927	2.9309	3.2432	2.5306	2.665
444	Terpenoids	Glaucarubolone 15-O-beta-D-glucopyranoside	5.3697	5.4973	5.3372	5.2451	5.2615	5.3662	5.5468	5.5319	5.6866	5.6189
445	Terpenoids	Armillane	5.0539	4.7911	4.6121	5.1547	4.8917	5.0294	4.9208	4.8362	4.7666	5.0053
446	Terpenoids	Musabalbisiane C	7.2785	6.9267	6.9185	6.6773	6.9762	7.0423	6.713	7.1736	7.0068	7.2833
447	Terpenoids	Crocin 4	4.152	5.3217	3.4875	3.3815	3.9251	4.3627	5.1853	3.3951	4.4224	4.3275
448	Terpenoid	Siderone	4.9862	4.7143	4.6964	4.7689	5.0408	5.1399	4.8701	4.7203	4.5181	4.8249

	s											
449	Terpenoids	Gibberellin A77	4.2319	2.968	3.2602	3.107	3.088	4.9343	3.049	2.9727	3.2372	3.85
450	Terpenoids	Methuyl tanshinonate	4.3781	5.4843	4.3386	3.741	4.5718	4.8346	5.4035	4.4035	4.8465	4.7079
451	Terpenoids	Phytocassane B	2.5739	2.5268	3.4721	2.6095	2.6146	4.9502	3.0078	2.5315	2.5311	3.0993
452	Terpenoids	Gibberellin A87	4.7896	5.3967	4.8488	4.6385	4.8707	4.9933	5.2655	4.7619	5.1974	5.1554
453	Terpenoids	Gibberellin A45	5.5397	5.4957	5.0403	5.0289	5.5034	5.5881	5.4119	5.5391	4.9543	5.3495
454	Terpenoids	Alpha-Tocopherolquinone	4.7671	4.9531	4.9486	4.9711	4.9958	4.9383	4.9185	4.9886	4.7866	4.9699
455	Terpenoids	Serratol	6.0794	5.9755	6.1241	6.2616	6.0388	5.9407	5.9452	6.4544	5.6503	5.8348
456	Terpenoids	Geranylcitronellol	3.7698	3.6459	3.3191	4.3178	3.7012	3.7026	3.674	3.1554	3.0475	3.709
457	Terpenoids	Yucalexin P21	6.225	5.9572	5.9476	6.0466	6.0292	5.9551	5.9772	5.9526	5.8763	5.8706
458	Terpenoids	Sterebin E	4.6984	4.7845	4.7312	4.686	4.8329	4.7581	4.6744	4.674	4.8348	4.6965
459	Terpenoids	Persicachrome	2.8424	2.8715	3.516	3.0593	3.4819	3.2387	3.2251	3.3812	3.348	2.9734
460	Terpenoids	Phytocassane D	4.962	4.9125	6.4687	4.8944	4.9347	5.3646	4.8969	4.9756	4.9313	4.7969
461	Terpenoid	Yucalexin A16	5.1827	5.2368	5.5842	5.2644	5.2601	5.115	5.2338	5.2651	5.2597	5.2388

	s											
462	Terpenoids	Cavipetin D	4.6659	4.0844	4.1106	4.8837	4.8058	5.1976	5.0358	2.159	2.1586	3.4399
463	Terpenoids	2-Acetoxy-3-geranylgeranyl-1,4-dihydroxybenzene	5.4261	5.2811	5.2687	5.573	5.5001	5.4267	5.3269	5.4332	5.255	5.3225
464	Terpenoids	Mangicrocin	5.7412	5.6245	5.5538	5.6602	5.5041	5.5678	5.5259	5.7369	5.8974	5.8196
465	Terpenoids	Musabalbisiane B	4.6686	5.7811	3.9223	4.5819	4.1408	5.1578	5.2729	3.7565	4.2345	4.3821
466	Terpenoids	Gibberellin A86	5.312	5.1144	4.9622	5.3342	5.2058	4.9361	5.1582	5.0545	5.0911	5.0455
467	Terpenoids	Pristanoylglycine	6.7244	5.9807	6.0891	6.6007	6.3193	6.5821	6.1485	5.9357	5.3005	6.0977
468	Terpenoids	3-hydroxypristanic acid	4.0341	4.2006	4.285	4.6133	4.4831	4.6219	4.2117	4.1927	3.8216	3.9275
469	Terpenoids	Sclareol	5.7639	5.0871	4.8479	3.7693	5.3197	6.159	5.6521	4.5059	4.1466	5.3661
470	Terpenoids	Sagittariol	6.0657	5.6339	4.9533	4.1462	5.9515	6.4385	5.8919	4.4563	4.3614	5.4375
471	Terpenoids	Austroinulin	3.6583	2.9505	2.9323	3.3215	2.9155	3.803	2.9907	2.8987	3.6167	3.2717
472	Terpenoids	Homophytanic acid	3.1776	3.1287	3.1721	3.1977	3.1941	3.2267	3.1804	3.1166	3.1324	3.1709
473	Terpenoids	Gibberellin A74	4.8012	4.8291	5.032	4.9932	4.7203	4.7349	4.6219	4.3312	4.6765	4.6608

474	Terpenoids	Musabalbisiane A	3.7319	3.8795	3.7015	3.5444	3.0482	3.6191	3.2633	3.3818	3.9008	3.6203
475	Terpenoids	Lactapiperanol D	4.574	4.3024	4.873	4.7881	4.3109	4.4419	4.4332	4.1691	4.0148	4.0641
476	Terpenoids	Antibiotic X 14889D	4.6427	3.6461	4.2453	4.1915	4.3225	4.5216	4.3768	4.0375	4.2938	4.0792
477	Terpenoids	Beta-Micropteroxanthin	3.7684	3.3376	3.5541	4.0659	3.4335	3.1501	3.3849	3.2409	3.0295	3.1622
478	Terpenoids	25-Acetylvulgaroside	0.437	1.3511	0.9239	5.6976	1.7487	2.3516	1.5119	0.3993	0.4088	0.4327
479	Terpenoids	(2Z,6E)-3,7,11,15,19-Pentamethyl-2,6-eicosadien-1-ol	4.9639	4.5106	3.9872	4.5798	5.1444	5.0573	5.0428	4.529	4.5748	4.642
480	Terpenoids	Lepidiumsesterterpenol	5.6405	5.8618	5.2481	6.0512	5.8706	5.8063	5.8076	5.7366	5.5124	5.5959
481	Terpenoids	(9E)-Valenciaxanthin	3.9182	3.1576	3.5392	2.9088	5.2738	4.7061	4.5164	4.6112	3.7883	4.4851
482	Terpenoids	Beta-Doradecin	2.5598	1.2792	4.6539	2.5999	4.6164	4.4993	3.8102	3.8654	3.6183	3.9121
483	Terpenoids	Ganodermatriol	5.6586	5.4322	5.4971	5.7241	6.3561	5.8763	5.9428	5.9202	5.5628	5.7381
484	Terpenoids	Hoduloside IX	6.6182	6.4468	6.3996	6.7271	6.4541	6.2241	6.2995	6.4712	6.334	6.3644
485	Terpenoids	Astragaloside IV	3.5648	3.6283	3.829	3.6005	3.869	3.6426	3.5987	3.5224	3.5906	3.5812
486	Terpenoid	Cauloside C	3.3121	3.4087	3.3067	3.6489	3.3287	3.3672	3.315	3.2512	3.2669	3.3055

	s											
487	Terpenoids	Pongamoside D	4.8011	5.2347	4.7537	4.5266	5.0796	5.0947	4.9855	5.0979	5.4376	5.1181
488	Lipids and lipid-like molecules	PE(16:0/0:0)	6.3995	6.3031	6.296	6.0792	6.537	6.2742	6.4558	6.4673	6.1908	6.2901
489	Lipids and lipid-like molecules	GPEtn(14:0/22:2)	7.1522	7.2357	7.1435	7.1995	7.2718	7.251	7.2343	7.3502	7.325	7.2999
490	Lipids and lipid-like molecules	LysoPE(0:0/18:4(6Z,9Z,12Z,15Z))	5.4541	5.4465	5.588	5.5231	5.3003	5.5867	5.611	4.9453	4.973	5.5573
491	Lipids and lipid-like molecules	GPEtn(15:0/20:3)	5.6404	5.838	5.9507	5.653	5.6422	5.7907	5.7194	5.6458	5.7102	5.4688
492	Lipids and lipid-lik	PE(DiMe(11,3)/DiMe(9,3))	2.4859	0.4599	2.8569	5.3236	2.6697	4.5651	4.5685	0.8421	4.3724	4.399

	e molecules											
493	Lipids and lipid-like molecules	GPEtn(14:1/22:2)	7.2122	7.3438	7.3068	7.232	7.2955	7.324	7.2935	7.3953	7.4645	7.3807
494	Lipids and lipid-like molecules	PE(20:4(8Z,11Z,14Z,17Z)/P-18:0)	3.1752	3.128	3.1375	3.2109	3.6439	3.253	3.5674	3.1328	3.3354	3.5711
495	Lipids and lipid-like molecules	LysoPE(0:0/18:2(9Z,12Z))	2.8923	3.9686	3.3974	2.3266	3.9636	3.374	2.3021	3.8544	4.3977	3.9913
496	Lipids and lipid-like molecules	LysoPE(22:1(13Z)/0:0)	5.5086	5.0166	5.1868	5.0187	5.2189	5.3082	5.2146	4.9924	5.0155	5.4114
497	Lipids and lipid-like e	GPEtn(16:0/18:1)	7.038	7.1017	6.9507	7.1364	7.1648	7.0764	7.1209	7.2676	6.9606	7.0114

	molecules											
498	Lipids and lipid-like molecules	PE(16:1(9Z)/20:2(11Z,14Z))	6.0739	6.1277	6.1073	5.8444	6.0856	6.1423	6.1253	6.0932	6.2708	6.1422
499	Lipids and lipid-like molecules	PE(16:0/20:5(5Z,8Z,11Z,14Z,17Z))	4.678	4.7763	4.7426	4.4567	5.0512	5.0063	5.0386	4.9227	4.8924	4.6602
500	Lipids and lipid-like molecules	PE(18:4(6Z,9Z,12Z,15Z)/22:1(13Z))	6.5509	6.43	6.5091	6.6314	6.5652	6.5608	6.5983	6.4735	6.6427	6.6951
501	Lipids and lipid-like molecules	PE(18:3(9Z,12Z,15Z)/P-16:0)	6.6805	6.8145	6.8885	6.4207	6.7699	6.8056	6.8144	6.8311	6.7304	6.6817
502	Lipids and lipid-like molecules	PE(20:0/18:2(9Z,12Z))	5.8958	5.9762	5.8979	5.8763	5.9799	6.4011	6.0046	6.0448	6.0587	6.0796

503	Lipids and lipid-like molecules	GPEtn(16:1/22:2)	5.5936	5.6662	5.6656	5.6129	5.7752	5.8496	5.7672	5.8058	5.8953	5.7206
504	Lipids and lipid-like molecules	PE(16:0/22:5(4Z,7Z,10Z,13Z,16Z))	5.9069	6.0072	6.0168	5.6677	6.0004	6.0805	6.0022	6.0592	6.1409	6.1225
505	Lipids and lipid-like molecules	PE(15:0/16:1(9Z))	6.7901	7.1896	7.1748	7.4011	7.1764	7.1901	7.3484	7.2223	7.2268	7.153
506	Lipids and lipid-like molecules	PE(14:0/16:1(9Z))	5.3933	5.8285	5.7439	5.5483	5.4913	5.5107	5.3475	5.2952	5.2386	5.1444
507	Lipids and lipid-like molecules	LysoPE(20:0/0:0)	6.141	4.9181	4.8071	5.0094	5.1225	5.9166	5.0946	5.0721	4.7165	5.4126
508	Lipids	LysoPE(18:2(9Z,12Z)/0:0	6.6971	6.5644	6.6623	6.5216	6.7451	6.5592	6.6344	6.6943	6.7166	6.7517

	and lipid-like molecules	)										
509	Lipids and lipid-like molecules	PE(18:3(6Z,9Z,12Z)/18:1(11Z))	5.9865	6.0347	6.0058	5.9544	5.9895	5.9067	6.0063	6.0314	6.0404	5.9496
510	Lipids and lipid-like molecules	LysoPE(0:0/15:0)	5.547	5.4256	5.6951	5.8662	5.4984	5.7069	5.46	5.5215	5.6097	5.5711
511	Lipids and lipid-like molecules	Hydroxyclomipramine	4.2908	4.3942	4.4856	4.4131	4.2694	4.5834	4.6948	4.1021	4.5457	4.2829
512	Lipids and lipid-like molecules	LysoPE(18:3(6Z,9Z,12Z)/0:0)	4.3643	4.5357	4.1694	4.242	4.4815	4.0717	4.2823	4.277	4.0274	2.8261
513	Lipids and	GPEtn(18:2/18:2)	7.0131	7.0627	7.1785	6.8817	7.0377	7.0871	7.0939	7.073	7.2852	7.0611

	lipid-like molecules											
514	Lipids and lipid-like molecules	GPEtn(18:2/18:3)	2.9442	2.7786	3.0171	3.5524	3.1177	3.7426	2.8303	2.7665	2.7823	2.8207
515	Lipids and lipid-like molecules	PE(18:3(9Z,12Z,15Z)/16:0)	5.6797	5.7439	5.709	5.6784	5.8681	5.9262	5.8082	5.8533	5.625	5.5062
516	Lipids and lipid-like molecules	GPEtn(18:1/18:1)	7.2462	7.283	7.2315	6.8958	7.2462	7.2285	7.2623	7.2609	7.3296	7.2952
517	Lipids and lipid-like molecules	PE(18:2(9Z,12Z)/20:1(11Z))	5.7649	5.7941	5.8831	5.6215	5.876	5.8779	5.8824	5.7923	6.0076	5.7913
518	Lipids and lipid-lik	PE(18:0/18:1(11Z))	6.2153	6.3322	6.012	5.7264	6.1834	6.0996	6.2275	6.3278	5.7902	5.9612

	e molecules											
519	Lipids and lipid-like molecules	PE(15:0/22:2(13Z,16Z))	5.4384	5.5373	5.7004	5.2497	5.4884	5.5527	5.5325	5.456	5.5165	5.2853
520	Lipids and lipid-like molecules	PE(18:1(11Z)/18:3(6Z,9Z,12Z))	6.5971	6.1576	6.2802	6.4329	6.4329	6.5224	6.3033	6.1255	6.2568	6.2781
521	Lipids and lipid-like molecules	GPEtn(18:1/18:2)	7.4539	7.4706	7.4714	7.1922	7.4151	7.4238	7.4287	7.4306	7.552	7.4698
522	Lipids and lipid-like molecules	PE(22:6(4Z,7Z,10Z,13Z,16Z,19Z)/P-18:1(11Z))	5.8708	5.9473	5.94	5.6705	5.8912	5.7843	5.8745	5.9024	6.0278	5.9506
523	Lipids and lipid-like	PE(16:0/18:3(6Z,9Z,12Z))	5.8391	5.6709	5.8194	5.7876	5.8676	5.791	5.732	5.6825	5.5369	5.4847

	molecules											
524	Lipids and lipid-like molecules	PE(16:0/18:2(9Z,12Z))	5.0456	5.1019	5.3286	4.9342	5.0648	5.0279	5.1623	5.0543	5.3159	4.8828
525	Lipids and lipid-like molecules	LysoPE(0:0/16:1(9Z))	5.0669	4.8396	4.872	4.803	5.571	5.4006	4.8679	5.0876	4.9079	5.2847
526	Lipids and lipid-like molecules	LysoPE(0:0/18:3(9Z,12Z,15Z))	5.6539	5.6364	5.455	5.4755	5.7206	5.4768	5.5096	5.7865	5.7367	5.7152
527	Lipids and lipid-like molecules	PE(18:0/0:0)	5.9162	5.5375	5.4774	6.1313	5.7876	5.6851	5.7636	5.7046	5.2355	5.6508
528	Lipids and lipid-like molecules	GPEtn(12:0/22:2)	4.7521	4.8073	5.122	4.8091	4.8346	4.9977	4.959	4.6772	5.0606	4.6649

529	Lipids and lipid-like molecules	GPEtn(10:0/26:1)	6.4294	6.5083	6.2469	6.7632	6.5657	6.5966	6.5791	6.6898	6.3546	6.5176
530	Lipids and lipid-like molecules	PE(16:1(9Z)/17:1(9Z))	4.718	5.0925	5.0924	4.5875	5.1275	5.1203	5.1608	5.2277	5.2145	4.7851
531	Lipids and lipid-like molecules	GPEtn(10:0/22:0)	5.057	5.2879	5.3635	5.0841	5.233	5.2784	5.3959	5.3226	5.1256	5.127
532	Lipids and lipid-like molecules	PE(19:1(9Z)/18:4(6Z,9Z,12Z,15Z))	5.6584	5.7225	6.9432	5.5994	5.61	5.5062	5.6092	5.6478	5.6311	5.5529
533	Lipids and lipid-like molecules	PE(20:1/0:0)	5.8127	5.7535	5.7027	4.7005	5.7314	5.7702	5.7405	5.6454	5.685	5.7465
534	Lipids	PE(18:1(9Z)/0:0)	5.6584	5.5622	5.6149	5.4108	5.6924	5.5203	5.5648	5.6357	5.6008	5.7199

	and lipid-like molecules											
535	Lipids and lipid-like molecules	PE(18:2/0:0)	5.4019	5.2683	5.3372	5.1895	5.48	5.3112	5.3189	5.3155	5.3563	5.3744
536	Lipids and lipid-like molecules	1-Palmitoyl-2-linoleoyl PE	7.1354	7.1991	7.1849	6.8865	7.1829	7.1618	7.2118	7.227	7.1865	7.0304
537	Lipids and lipid-like molecules	PE(16:1/0:0)	4.0793	4.1715	4.2515	4.5958	4.9392	4.3652	4.4437	4.6137	4.5384	4.0316
538	Lipids and lipid-like molecules	PC(18:1(11Z)/18:2(9Z,12Z))	8.6074	8.7126	8.7264	8.2692	8.6912	8.6799	8.6702	8.7026	8.7876	8.6899
539	Lipids and	PC(18:1(11Z)/18:3(6Z,9Z,12Z))	8.1478	8.2752	8.4054	7.9598	8.2187	8.2946	8.2824	8.2487	8.4901	8.2197

	lipid-like molecules										
540	Lipids and lipid-like molecules	PC(18:3/0:0)	5.5337	5.5581	5.6778	5.284	5.7198	5.6562	5.7124	5.6296	5.5496	5.6154
541	Lipids and lipid-like molecules	PC(16:0/18:3(9Z,12Z,15Z))	6.2591	6.5171	6.7023	5.8509	6.3381	6.4307	6.4759	6.4442	6.5651	6.252
542	Lipids and lipid-like molecules	PC(18:3(9Z,12Z,15Z)/20:0)	6.5533	6.793	6.7836	4.8257	6.6114	6.5251	6.5447	6.7018	6.584	6.2118
543	Lipids and lipid-like molecules	1-Linoleoylglycerophosphocholine	5.8356	5.6535	5.7959	5.3794	5.7052	5.5348	5.6358	5.6751	5.6884	5.7857
544	Lipids and lipid-lik	GPCho(18:2/18:2)	6.6374	6.7454	6.8481	6.4551	6.5633	6.6097	6.6158	6.6164	6.7002	6.4292

	e molecules											
545	Lipids and lipid-like molecules	LysoPC(18:1(9Z))	5.4142	5.2013	5.1986	5.0152	5.3654	5.0161	5.208	5.2292	5.1874	5.5012
546	Lipids and lipid-like molecules	GPCho(18:1/18:4)	7.1951	7.2329	7.254	7.2657	7.2619	7.3106	7.3137	7.2655	7.2597	7.2681
547	Lipids and lipid-like molecules	PC(14:0/16:0)	5.6254	5.1473	5.3255	4.9313	5.3461	5.2666	5.4247	5.2017	4.3105	5.219
548	Lipids and lipid-like molecules	PC(15:0/20:3(8Z,11Z,14Z))	5.8387	5.8859	6.0225	5.3865	5.8921	6.1254	5.9594	5.8907	6.003	5.8397
549	Lipids and lipid-like	LysoPC(18:1(11Z))	4.8254	4.5974	4.3274	3.938	4.6739	4.0052	4.2197	4.5127	4.4408	4.6795

	molecules											
550	Lipids and lipid-like molecules	2-Palmitoylglycerophosphocholine	4.2319	3.9778	3.8843	3.9023	4.208	4.1629	3.9147	3.685	3.7864	4.3162
551	Lipids and lipid-like molecules	PC(18:1(11Z)/18:4(6Z,9Z,12Z,15Z))	6.4185	6.463	6.5952	6.5536	6.5101	6.5727	6.5535	6.4102	6.549	6.3132
552	Lipids and lipid-like molecules	PC(15:0/22:4(7Z,10Z,13Z,16Z))	4.5642	4.9302	4.9788	4.7382	4.7516	4.7672	4.897	4.7774	5.4987	4.6006
553	Lipids and lipid-like molecules	GPCho(14:0/20:1)	7.5503	7.5036	7.4374	7.2795	7.3873	7.4994	7.503	7.4974	7.4791	7.4273
554	Lipids and lipid-like molecules	LysoPC(O-18:0)	4.824	4.8681	4.8353	5.5935	5.1401	5.0582	4.7784	5.1702	4.5232	4.5708

555	Lipids and lipid-like molecules	LysoPC(16:1(9Z))	5.8385	5.841	5.9133	5.6058	6.1055	5.7617	5.899	6.0213	5.6671	5.9334
556	Lipids and lipid-like molecules	PC(18:0/18:3(9Z,12Z,15Z))	7.5018	7.5025	7.4489	7.6129	7.5483	7.4837	7.5144	7.5226	7.5846	7.5609
557	Lipids and lipid-like molecules	LysoPC(18:0)	5.7077	5.4761	5.3919	5.09	5.5196	5.3472	5.5341	5.365	4.9012	5.3512
558	Lipids and lipid-like molecules	LysoPC(18:2(9Z,12Z))	4.4461	3.3855	3.5487	4.3667	3.8431	3.5817	3.3222	3.2168	1.4452	4.1109
559	Lipids and lipid-like molecules	PC(16:0/18:3(6Z,9Z,12Z))	5.0015	5.2231	5.4468	4.6711	5.0983	5.0285	5.2658	5.13	5.1136	4.9478
560	Lipids	LysoPC(15:0)	5.5186	5.4995	5.5161	5.1707	5.5577	5.3306	5.532	5.5692	5.417	5.455

	and lipid-like molecules											
561	Lipids and lipid-like molecules	PC(18:0/18:2(9Z,12Z))	8.0045	8.1716	8.0664	7.6624	7.7781	7.8752	8.0158	8.1033	8.1484	8.0064
562	Lipids and lipid-like molecules	Glycerophosphocholine	6.4578	6.2736	6.3023	6.2273	6.2826	6.0319	6.3234	6.3444	6.0859	6.1611
563	Lipids and lipid-like molecules	PC(16:0/18:2(9Z,12Z))	6.3982	6.6228	6.5351	6.8676	6.588	6.399	6.6624	6.6416	6.5344	6.4386
564	Lipids and lipid-like molecules	PC(16:0/14:1(9Z))	5.2763	5.3088	5.3438	5.2704	5.6491	5.2355	5.465	5.4162	5.2695	5.0961
565	Lipids and	PC(18:2(9Z,12Z)/18:3(6Z,9Z,12Z))	4.439	4.733	5.2883	2.4704	4.5614	4.8619	4.8965	4.4437	5.0311	4.5101

	lipid-like molecules										
566	Lipids and lipid-like molecules	PC(14:0/24:1(15Z))	5.9603	5.8407	6.0592	6.3257	6.0641	6.0047	6.0881	5.7757	5.7213	5.9621
567	Lipids and lipid-like molecules	PC(16:0/22:2(13Z,16Z))	5.9426	5.9987	6.112	5.9729	5.9993	5.9096	6.0186	5.9729	6.0971	6.0923
568	Lipids and lipid-like molecules	LysoPC(20:1(11Z))	5.3613	5.2757	5.3248	4.6869	5.3762	4.8489	5.3312	5.2354	5.1941	5.1985
569	Lipids and lipid-like molecules	PC(16:0/18:1(11Z))	7.2263	7.3841	7.2867	7.2083	7.2967	7.1962	7.3279	7.4111	7.3183	7.2057
570	Lipids and lipid-lik	LysoPC(18:3(6Z,9Z,12Z))	3.0403	1.7783	3.0102	1.7968	2.6329	2.3099	2.8493	2.6933	2.8096	2.0464

	e molecules											
571	Lipids and lipid-like molecules	PC(18:2/0:0)	7.8902	7.7586	7.8547	7.5727	7.8984	7.6585	7.8047	7.8525	7.8455	7.8845
572	Lipids and lipid-like molecules	PC(16:0/22:5(4E,7E,10E,13E,16E))[U]	7.7449	7.8344	7.7625	6.4132	7.8401	7.7008	7.7587	7.8467	7.8252	7.7771
573	Lipids and lipid-like molecules	PC(16:0/0:0)[U]	7.6326	7.4393	7.516	7.2616	7.6813	7.443	7.5488	7.5597	7.2818	7.4942
574	Lipids and lipid-like molecules	PC(18:1/0:0)	8.0615	7.8933	7.9066	7.6516	8.0929	7.8064	7.9281	7.9774	7.8796	8.0898
575	Lipids and lipid-like	PC(18:0/22:6(4E,7E,10E,13E,16E,19E))[U]	5.867	6.0465	6.1366	4.9148	5.9999	5.8923	5.9581	6.0299	6.0071	5.6957

	molecules											
576	Lipids and lipid-like molecules	PC(P-16:0/2:0)	6.6	6.3486	6.3331	6.033	6.5223	6.314	6.3835	6.3884	6.3669	6.5322
577	Lipids and lipid-like molecules	1,2-dipalmitoyl-sn-glycero-3-PC	7.7537	7.5337	7.466	7.5362	7.5103	7.4934	7.5281	7.4364	7.0188	7.257
578	Lipids and lipid-like molecules	PC(20:2(11Z,14Z)/0:0)	5.763	5.527	5.6195	5.2888	5.8626	5.5204	5.6485	5.5138	5.5796	5.8314
579	Lipids and lipid-like molecules	LPA(18:2(9Z,12Z)/0:0)	5.9746	5.2892	5.4905	6.0709	5.4833	5.4069	5.2004	5.2601	4.7246	5.2817
580	Lipids and lipid-like molecules	PA(16:0/20:3(8Z,11Z,14Z))	5.672	5.383	5.3064	6.2517	5.6471	5.5131	5.3743	5.6263	5.3866	5.5948

581	Lipids and lipid-like molecules	1-(4Z,7Z,10Z,13Z,16Z,19Z-docosahexaenoyl)-glycero-3-phosphate	5.3757	5.28	5.4192	5.2618	5.5172	5.1924	5.3562	5.4449	5.5036	5.4654
582	Lipids and lipid-like molecules	1-(5Z,8Z,11Z,14Z,17Z-eicosapentaenoyl)-glycero-3-phosphate	5.209	4.1954	3.2544	4.6654	4.4059	3.4787	4.7542	3.8335	3.4189	3.3159
583	Lipids and lipid-like molecules	LysoPA(8:0/0:0)	6.356	6.2759	6.0811	6.392	6.184	5.9807	6.2266	6.1273	6.2971	6.3956
584	Lipids and lipid-like molecules	LysoPA(0:0/18:2(9Z,12Z))	5.3348	4.6809	4.8043	5.7081	4.9301	4.7413	4.6719	4.8032	3.9013	4.5609
585	Lipids and lipid-like molecules	PA(16:0/20:5(5Z,8Z,11Z,14Z,17Z))	5.2468	4.8742	5.0164	6.0226	5.3267	5.1443	5.0988	5.3679	4.7262	5.1646
586	Lipids	PA(16:0/18:1(11Z))	5.4458	5.2715	4.8402	5.7479	5.552	5.4215	5.1777	5.2268	5.0565	5.2636

	and lipid-like molecules										
587	Lipids and lipid-like molecules	PA(18:1(9Z)/20:1(11Z))	5.9771	5.9564	5.7661	5.7637	5.848	6.4341	5.8331	5.9629	6.0616	6.0126
588	Lipids and lipid-like molecules	1-(11Z,14Z-eicosadienoyl)-glycero-3-phosphate	5.1535	3.2954	3.1363	3.9163	3.7492	3.9977	3.1837	3.3337	3.6356	4.6822
589	Lipids and lipid-like molecules	1-(11Z-eicosenoyl)-glycero-3-phosphate	5.2419	3.5454	3.6707	4.1474	4.1549	4.3687	3.6162	3.7806	3.9433	4.8098
590	Lipids and lipid-like molecules	1,2-dioleoyl-sn-Glycero-3-Phosphate	5.9352	5.7318	5.6326	6.2443	6.0101	6.0136	5.9392	6.0468	5.6066	5.8124
591	Lipids and	PA(14:0/0:0)	3.9623	3.0491	3.0659	4.4032	3.6572	3.1205	3.1952	3.3012	3.0262	3.0647

	lipid-like molecules											
592	Lipids and lipid-like molecules	PA(15:0/18:2(9Z,12Z))	4.9938	4.6189	4.6615	5.5769	4.9905	4.7218	4.7998	5.1849	4.5171	4.6553
593	Lipids and lipid-like molecules	PA(16:1(9Z)/22:0)	4.6555	4.3338	4.5253	3.9773	4.3512	4.4163	4.5062	4.1367	4.4864	4.4549
594	Lipids and lipid-like molecules	PA(18:2(9Z,12Z)/18:3(6Z,9Z,12Z))	0.2492	1.2969	0.2468	4.0945	2.4503	0.2714	1.247	3.2593	0.2301	0.2463
595	Lipids and lipid-like molecules	PA(20:2(11Z,14Z)/18:2(9Z,12Z))	5.5624	4.4439	4.43	4.0818	3.6091	6.2321	4.9104	3.9517	3.4235	5.3312
596	Lipids and lipid-lik	PA(18:2(9Z,12Z)/20:0)	6.0711	5.7978	5.5901	6.6247	5.9769	6.0232	5.8312	5.868	5.6289	5.746

	e molecules										
597	Lipids and lipid-like molecules	PA(16:1(9Z)/16:0)	5.1072	4.6096	4.5223	5.6286	4.9997	4.7265	4.69	5.0371	4.3833	4.6659
598	Lipids and lipid-like molecules	PA(20:1(11Z)/22:2(13Z,16Z))	4.8069	5.0044	5.1266	4.656	5.0269	4.7331	4.9211	5.026	5.4574	5.1239
599	Lipids and lipid-like molecules	PA(16:0/18:3(6Z,9Z,12Z))	5.5162	5.2149	5.1882	5.9962	5.2841	5.0347	5.1636	5.4055	4.8571	5.0853
600	Lipids and lipid-like molecules	PA(14:0/18:2(9Z,12Z))	4.8761	4.2212	4.3541	5.6035	4.7401	4.3495	4.4213	4.7832	3.9334	4.2094
601	Lipids and lipid-like	PA(18:1(9Z)/18:3(6Z,9Z,12Z))	5.0455	4.8262	4.7833	5.3911	5.7951	5.1806	5.0052	5.3111	5.5883	5.7182

	molecules											
602	Lipids and lipid-like molecules	1-(9Z-hexadecenoyl)-glycero-3-phosphate	3.854	3.281	3.1979	4.9236	4.0672	3.4522	3.489	4.5687	3.1775	3.4289
603	Lipids and lipid-like molecules	1-(8Z,11Z,14Z-eicosatrienoyl)-glycero-3-phosphate	4.2272	2.97	2.8527	4.489	3.4037	3.6206	3.2948	2.9012	3.1451	3.2397
604	Lipids and lipid-like molecules	1-pentadecanoyl-glycero-3-phosphate	5.4803	4.1694	2.351	2.0082	3.8737	4.379	3.0846	3.9693	4.6112	5.2755
605	Lipids and lipid-like molecules	Glycerol 3-phosphate	5.6299	4.894	5.0231	6.0416	5.1628	5.229	5.0049	4.9757	4.5923	5.214
606	Lipids and lipid-like molecules	PA(17:1(9Z)/17:1(9Z))	5.7231	5.3385	5.189	6.3235	5.6524	5.4429	5.3475	5.5229	4.879	5.1623

607	Lipids and lipid-like molecules	PA(18:2(9Z,12Z)/18:2(9Z,12Z))[U]	5.9274	5.5642	5.5574	6.4797	5.7425	5.5542	5.5126	5.6993	5.3462	5.5473
608	Lipids and lipid-like molecules	PA(14:1(9Z)/0:0)	6.5888	6.5929	6.6082	6.8146	6.5966	6.8616	6.557	6.4874	6.537	6.5363
609	Lipids and lipid-like molecules	13(S)-HOTrE	6.7999	6.5231	6.5492	6.6864	6.9429	6.9065	6.8	6.4897	6.4766	6.7992
610	Lipids and lipid-like molecules	(+/-)13-HpODE	4.9955	5.0654	4.8973	5.1156	4.9676	4.9454	4.8408	4.723	4.6733	4.7321
611	Lipids and lipid-like molecules	13S-HpOTrE(gamma)	5.6759	5.5213	6.1582	5.9276	5.7947	6.7966	5.8754	5.7511	5.2997	5.8196
612	Lipids	PI(18:2(9Z,12Z)/0:0)	6.167	5.963	6.1088	6.3953	6.1674	5.6398	5.9997	6.1047	5.9231	6.3433

	and lipid-like molecules											
613	Lipids and lipid-like molecules	PI(16:0/20:3(8Z,11Z,14Z))	5.8551	5.8271	5.802	5.9861	5.8641	5.8394	5.8635	5.8021	5.8788	5.8725
614	Lipids and lipid-like molecules	PI(16:2(9Z,12Z)/18:0)	6.321	6.3121	6.3306	6.4755	6.4266	6.4164	6.437	6.3728	6.4175	6.3614
615	Lipids and lipid-like molecules	1-Oleoylglycerophosphoinositol	5.6096	5.3768	5.3177	5.7187	5.4595	5.046	5.3522	5.4415	5.2099	5.8079
616	Lipids and lipid-like molecules	PI(18:0/22:5(7Z,10Z,13Z,16Z,19Z))	3.996	3.9632	3.8929	3.6406	4.0684	4.2223	3.8724	3.67	3.4287	3.6077
617	Lipids and	PI(16:0/22:4(10Z,13Z,16Z,19Z))	3.8921	4.6534	3.9815	3.6433	3.9123	3.6963	3.9407	4.4108	3.9642	3.5624

	lipid-like molecules										
618	Lipids and lipid-like molecules	PI(18:0/18:2(9Z,12Z))	7.6616	7.7282	7.6166	7.5965	7.6674	7.5966	7.7062	7.697	7.6573	7.6524
619	Lipids and lipid-like molecules	1-Palmitoylglycerophosphoinositol	6.5556	6.4207	6.4695	6.6855	6.5369	6.2288	6.455	6.4884	6.2884	6.6
620	Lipids and lipid-like molecules	1-Stearoylglycerophosphoinositol	6.1608	5.9852	5.9101	6.2334	6.0351	5.716	6.0114	5.9222	5.7485	6.1451
621	Lipids and lipid-like molecules	PI(16:0/18:2(9Z,12Z))	8.0499	8.1017	8.0045	8.0262	8.0862	8.005	8.0627	8.1275	8.0546	7.9773
622	Lipids and lipid-lik	PI(16:0/16:0)	6.3501	6.3121	6.3823	6.303	6.3159	6.2952	6.4075	6.3402	6.197	6.2712

	e molecules											
623	Lipids and lipid-like molecules	1-(sn-Glycero-3-phospho)-1D-myo-inositol	6.3766	6.4274	6.5372	6.249	6.5888	6.295	6.4399	6.9422	6.5857	6.1841
624	Lipids and lipid-like molecules	PI(16:1(9Z)/22:4(7Z,10Z,13Z,16Z))	4.0082	4.0023	4.138	3.9834	3.7976	3.8106	3.8555	4.427	4.5602	4.0663
625	Lipids and lipid-like molecules	PI(14:1(9Z)/0:0)	5.3866	5.5594	5.9448	5.8158	5.5244	5.4354	5.6934	5.4713	5.3474	5.3814
626	Lipids and lipid-like molecules	PG(O-20:0/0:0)	3.934	3.6847	4.3573	4.6367	4.9222	5.441	4.5736	4.5346	4.7706	5.4095
627	Lipids and lipid-like e	GPIns(18:2/18:2)	7.9324	7.9279	7.9098	7.925	7.8854	7.8726	7.9088	7.9135	7.9078	7.9015

	molecules											
628	Lipids and lipid-like molecules	PG(16:0/0:0)[U]	7.1945	6.5997	6.5401	6.6875	6.7178	6.7529	6.5844	6.7466	6.5661	7.0294
629	Lipids and lipid-like molecules	Cer(d16:2(4E,6E)/20:0(2OH))	6.7682	5.892	6.2569	6.5454	6.5763	6.569	6.3715	6.11	5.6265	6.117
630	Lipids and lipid-like molecules	Cer(d16:1(4E)/24:0(2OH))	5.836	5.4089	5.4256	6.0617	5.5119	5.7695	5.5873	5.6191	5.3251	5.0449
631	Lipids and lipid-like molecules	Cer(t18:0/22:0(2OH))	5.0485	5.0792	5.0601	5.2106	5.2402	5.314	5.1515	4.8165	4.8055	5.0546
632	Lipids and lipid-like molecules	Cer(t18:0/22:0)	5.4658	5.1873	5.152	5.6058	5.4242	5.5332	5.336	5.2971	5.2939	4.9902

633	Lipids and lipid-like molecules	Cer(d16:1(4E)/22:0(2OH))	5.2543	4.4147	4.5968	5.3501	5.0373	5.0242	4.6476	4.7014	4.0478	4.0472
634	Lipids and lipid-like molecules	Cer(t18:0/18:0)	5.6774	4.9129	4.9269	5.7866	5.7216	5.3891	5.321	5.1113	4.2559	4.759
635	Lipids and lipid-like molecules	Cer(d16:1(4E)/20:0(2OH))	5.6687	4.6227	5.2218	5.462	5.8153	5.5661	5.4509	5.3132	4.5785	5.3527
636	Lipids and lipid-like molecules	Cer(d18:0/16:0(2OH))	5.829	5.216	5.4546	6.0351	6.0627	5.6798	5.5786	5.5539	4.7996	5.2387
637	Lipids and lipid-like molecules	Cer(d16:2(4E,6E)/20:1(11Z)(2OH))	6.8675	6.0029	6.2487	6.6873	6.498	6.5751	6.2475	6.018	5.6212	5.9572
638	Lipids	Cer(d16:1(4E)/18:1(9Z)(	5.7123	5.6523	5.7394	5.8915	5.8048	5.7509	5.7354	5.709	5.612	5.4728

	and lipid-like molecules	2OH))									
639	Lipids and lipid-like molecules	PG(a-13:0/i-12:0)	5.8905	6.2515	6.0351	6.168	6.0245	6.0099	6.1612	5.9056	5.9519	5.8593
640	Lipids and lipid-like molecules	PG(18:1(11Z)/18:3(9Z,12Z,15Z))	6.292	6.0187	6.2635	6.22	6.0739	6.2776	6.1412	6.0575	6.3085	6.2431
641	Lipids and lipid-like molecules	PG(a-13:0/i-16:0)	1.2563	0.2338	0.2524	3.7506	2.473	0.2774	1.1447	2.8153	2.7313	2.0287
642	Lipids and lipid-like molecules	PG(18:1(11Z)/18:2(9Z,12Z))	5.9177	5.898	6.051	5.9539	5.8686	5.9955	5.8113	5.9005	6.3124	6.0938
643	Lipids and	PG(i-14:0/i-16:0)	4.5083	4.585	4.4891	3.6781	4.7862	4.4962	4.5322	4.8342	4.1928	4.1675

	lipid-lik e molecules											
644	Lipids and lipid-lik e molecules	1-Stearoylglycerophosph oglycerol	4.2336	4.4747	3.7226	3.3803	4.3052	4.5655	4.5468	4.1592	4.508	4.2122
645	Lipids and lipid-lik e molecules	PG(22:6(4Z,7Z,10Z,13Z,1 6Z,19Z)/22:6(4Z,7Z,10Z, 13Z,16Z,19Z))	0.6206	0.5838	0.6164	1.9704	1.5796	0.6585	0.6466	0.575	0.5866	0.6155
646	Lipids and lipid-lik e molecules	1-Oleoylglycerophosphos erine	4.5005	4.5532	4.6091	4.1143	4.0055	3.7766	4.3755	4.5656	4.6785	4.4792
647	Lipids and lipid-lik e molecules	1-Stearoylglycerophosph oserine	4.4535	4.5966	4.3016	3.8828	4.3522	3.6251	4.4312	4.6283	4.6966	4.6583
648	Lipids and lipid-lik	PS(22:2(13Z,16Z)/15:0)	5.6058	5.6269	5.6591	5.6664	5.6068	5.4722	5.5739	5.6813	5.6606	5.5134

	e molecules											
649	Lipids and lipid-like molecules	PS(18:0/18:1(9Z))	5.8073	5.8289	5.8113	5.4897	5.7999	5.7802	5.8363	5.7747	5.8809	5.8798
650	Lipids and lipid-like molecules	PS(16:0/18:1(9Z))	5.9255	6.003	5.9974	5.8166	5.9681	5.9349	5.9832	5.9976	5.9645	5.8634
651	Lipids and lipid-like molecules	PS(18:1(9Z)/18:2(9Z,12Z))	5.5917	5.708	5.796	5.5044	5.6509	5.5375	5.7232	5.6969	5.897	5.6391
652	Lipids and lipid-like molecules	PIP(16:0/18:0)	4.3972	4.696	5.1165	4.1946	4.3994	4.4257	3.6831	5.0364	3.9803	3.9983
653	Lipids and lipid-like	PGP(16:0/22:6(4Z,7Z,10Z,13Z,16Z,19Z))	3.7271	3.6763	3.9876	3.9392	3.6223	3.7535	3.7798	3.4526	3.6884	3.5554

	molecules										
654	Lipids and lipid-like molecules	CL(8:0/8:0/8:0/11:0)	4.5751	4.5804	4.672	4.5345	4.4617	4.2044	4.4114	4.7742	4.4696	4.4732
655	Lipids and lipid-like molecules	15(R),19(R)-hydroxy Prostaglandin E1	6.9438	6.4237	6.2805	6.8895	6.8272	7.0792	6.814	6.3775	6.0656	6.6153
656	Lipids and lipid-like molecules	17,20-dimethyl Prostaglandin F1alpha	4.4607	3.6912	3.8889	4.5918	4.3927	4.6606	4.2199	3.6825	3.4673	4.0198
657	Lipids and lipid-like molecules	9S,11R,15S-trihydroxy-2,3-dinor-13E-prostaenoic acid-cyclo[8S,12R]	4.7147	4.7285	5.4772	5.3335	4.8619	5.6312	5.1088	4.9458	4.4326	4.9978
658	Lipids and lipid-like molecules	Prostaglandin D1 Alcohol	4.0316	3.5347	3.625	3.2275	4.5631	4.8859	3.968	4.2036	4.4972	4.5056

659	Lipids and lipid-like molecules	2,3-dinor Prostaglandin E1	5.6738	5.6503	6.0312	5.9261	5.8788	6.2091	5.9289	5.8132	6.1056	6.226
660	Lipids and lipid-like molecules	Eriojaposide B	3.4324	3.7574	3.839	3.9129	3.5227	3.9917	3.7925	3.4993	3.9397	3.8482
661	Lipids and lipid-like molecules	(3b,9R)-5-Megastigmene-3,9-diol 9-[apiosyl-(1->6)-glucoside]	3.035	2.9878	3.1137	3.0706	3.2996	3.1127	3.0688	2.9925	2.9921	3.0513
662	Lipids and lipid-like molecules	Prenyl glucoside	4.048	4.1028	4.0893	4.1447	4.1869	4.0595	4.1666	4.039	3.6838	3.9066
663	Lipids and lipid-like molecules	(x)-2-Heptanol glucoside	2.8068	2.7597	2.8808	2.8468	3.0044	2.9152	2.8407	2.7829	2.9827	3.7343
664	Lipids	Xi-3-Hydroxy-5-phenylpe	3.6527	0.3994	2.7897	0.4512	0.4545	0.7165	0.45	0.4023	0.402	0.4387

	and lipid-like molecules	ntanoic acid O-beta-D-Glucopyranoside										
665	Lipids and lipid-like molecules	Ethyl 3-hydroxyoctanoate O-[glucosyl-(1->6)-glucoside]	3.6917	3.6445	3.654	3.7274	3.7325	3.7695	3.7256	3.6493	3.6488	3.7081
666	Lipids and lipid-like molecules	[6]-Gingerdiol 5-O-beta-D-glucopyranoside	5.0332	4.9003	5.01	4.9892	4.9915	5.0963	4.8524	4.8511	4.8659	5.0942
667	Lipids and lipid-like molecules	(S)-Nerolidol 3-O-[a-L-rhamnopyranosyl-(1->2)-b-D-glucopyranoside]	4.468	4.4208	4.4303	4.5037	4.5088	4.5458	4.5019	4.4256	4.4251	4.4844
668	Lipids and lipid-like molecules	Eriojaposide A	3.7451	3.7105	3.9069	4.289	3.6095	3.4209	3.8236	3.687	3.9746	3.6284
669	Lipids and	(3S,7E,9S)-9-Hydroxy-4,7-megastigmadien-3-one	4.8087	3.2773	3.6153	5.0082	4.7381	3.7575	4.6481	3.991	3.0442	3.1034

	lipid-like molecules	9-glucoside									
670	Lipids and lipid-like molecules	Isopentyl gentiobioside	3.5998	3.7195	4.4344	4.3106	4.6682	3.6392	4.3462	3.3322	4.56	4.3097
671	Lipids and lipid-like molecules	Dihydrozeatin-O-glucoside	6.065	5.255	5.718	5.4729	5.6628	5.3353	5.8839	5.8688	5.6596	5.8511
672	Lipids and lipid-like molecules	Sarmentosin	4.3076	3.9496	4.1192	4.5712	3.9697	4.3727	4.232	3.9908	3.7148	4.4146
673	Lipids and lipid-like molecules	Methyl helianthenoate A glucoside	5.693	5.9821	5.9358	5.908	5.7354	5.6737	5.8869	5.9362	6.0396	6.4619
674	Lipids and lipid-lik	Kojibiose	5.3481	4.8955	5.3338	4.6254	5.0029	5.1164	5.4632	4.8781	5.198	5.3029

	e molecules											
675	Lipids and lipid-like molecules	Butyl (S)-3-hydroxybutyrate [arabinosyl-(1->6)-glucoside]	4.2495	4.9759	4.4951	3.8016	4.647	3.8685	4.253	4.5489	4.4211	4.4937
676	Lipids and lipid-like molecules	Alpha-Ionol O-[arabinosyl-(1->6)-glucoside]	3.7831	4.3585	4.8003	4.3121	4.2152	4.338	4.4923	4.3541	4.5562	4.4331
677	Lipids and lipid-like molecules	7,8-Dihydro-3b,6a-dihydroxy-alpha-ionol 9-[apiosyl-(1->6)-glucoside]	5.413	5.6618	5.138	5.1996	5.5399	5.6216	5.5572	5.2762	5.6586	5.6026
678	Lipids and lipid-like molecules	7,8-Dihydrovomifoliol 9-[apiosyl-(1->6)-glucoside]	4.5878	4.9378	4.891	4.8683	4.5817	4.4164	4.7677	4.6733	4.7503	5.1472
679	Lipids and lipid-like e	Ethyl 7-epi-12-hydroxyjasmonate glucoside	4.6933	4.0907	4.1431	4.0679	5.4293	5.0554	4.6659	4.8756	4.9226	5.1666

	molecules										
680	Lipids and lipid-like molecules	(2E,4E,7R)-2,7-Dimethyl-2,4-octadiene-1,8-diol 8-O-b-D-glucopyranoside	4.5295	4.6199	4.8976	4.554	4.6519	4.5613	4.3449	4.7811	4.7872	4.9862
681	Lipids and lipid-like molecules	5,7-Megastigmadien-9-ol glucoside	5.1437	4.0798	4.4161	3.7248	4.2279	4.4915	4.0081	4.2205	4.6086	5.0856
682	Lipids and lipid-like molecules	Ilicifolinoside A	5.7074	5.6753	5.9326	5.8218	5.6678	5.6846	5.7229	5.7001	5.8798	5.7785
683	Lipids and lipid-like molecules	3-b-Galactopyranosyl glucose	3.8987	4.6878	4.5915	3.3739	4.007	3.7167	4.6533	4.623	5.0998	4.746
684	Lipids and lipid-like molecules	1-O-alpha-D-Glucopyranosyl-D-mannitol	5.6345	5.5589	5.707	5.4775	5.6315	5.5335	5.6515	5.6362	5.6048	5.6701

685	Lipids and lipid-like molecules	Ethyl (S)-3-hydroxybutyrate glucoside	2.7481	2.8349	2.7227	3.2779	2.9693	2.8355	2.6934	2.5575	2.6964	2.6827
686	Lipids and lipid-like molecules	Lactosamine	4.9686	5.0454	5.1141	5.0529	4.9846	4.8208	4.9576	5.094	5.0016	4.8279
687	Lipids and lipid-like molecules	4-(Methylnitrosamino)-1-(3-pyridyl)-1-butanol glucuronide	5.5106	5.464	5.3297	5.5169	5.3822	5.2356	5.4637	5.3634	5.4371	5.4666
688	Lipids and lipid-like molecules	(2R,6x)-7-Methyl-3-methylene-1,2,6,7-octanetetrol 2-glucoside	2.8946	2.9323	3.7055	3.122	3.7199	3.3166	3.1643	2.7512	3.4249	2.9688
689	Lipids and lipid-like molecules	Osmaronin	4.5022	4.2516	4.3871	4.9924	4.6037	4.2004	4.4068	3.9314	4.3875	4.9652
690	Lipids	Todatriol glucoside	4.8712	4.5843	4.8534	5.1338	4.756	4.6438	4.6306	4.676	4.2719	4.3086

	and lipid-like molecules											
691	Lipids and lipid-like molecules	1-Octen-3-yl glucoside	5.1759	4.9244	4.7519	4.979	5.1894	5.4296	5.1266	4.498	4.5238	4.5798
692	Lipids and lipid-like molecules	(R)-1-O-[b-D-Glucopyranosyl-(1->6)-b-D-glucopyranoside]-1,3-octanediol	3.6966	4.6329	3.658	3.2984	3.9975	4.7396	3.7946	3.2174	4.9542	5.5123
693	Lipids and lipid-like molecules	(R)-1-O-b-D-glucopyranosyl-1,3-octanediol	5.827	5.6121	5.522	5.8278	5.8747	5.8939	5.8514	5.4774	5.4857	5.4835
694	Lipids and lipid-like molecules	6-Epi-7-isocucurbic acid glucoside	4.0885	4.2472	4.4907	4.272	4.6195	4.8381	4.0756	4.6462	4.871	5.0855
695	Lipids and	Palmitoyl glucuronide	5.0673	4.297	4.4113	4.1616	4.3368	5.1599	4.6632	3.9899	4.4682	4.609

	lipid-like molecules										
696	Lipids and lipid-like molecules	7,8-Dihydro-3b,6a-dihydroxy-alpha-ionol 9-glucoside	4.4492	4.529	4.5341	4.0618	5.1097	4.698	4.6371	4.3297	4.8339	4.8693
697	Lipids and lipid-like molecules	Hexyl glucoside	5.354	5.4068	5.4445	5.3741	5.5036	5.3949	5.4135	5.3088	5.4047	5.3808
698	Lipids and lipid-like molecules	(3S,7E,9R)-4,7-Megastigmadiene-3,9-diol 9-[apiosyl-(1->6)-glucoside]	4.7908	5.1346	4.3762	4.436	4.9539	5.1011	5.0584	4.6956	5.1744	5.0479
699	Lipids and lipid-like molecules	Methyl (3x,4E,10R)-3,10-dihydroxy-4,11-dodecadiene-6,8-diynoate 10-glucoside	4.1597	4.3073	4.5271	4.6865	4.2444	4.5077	4.5703	3.9944	4.3153	4.1449
700	Lipids and lipid-like	(S)-3-Octanol glucoside	3.9767	4.2562	5.0983	4.8795	4.2564	5.1258	4.4842	4.4671	3.5805	4.4318

	e molecules											
701	Lipids and lipid-like molecules	1-(beta-D-Glucopyranosyloxy)-3-octanone	4.483	4.5194	4.6765	5.1061	5.05	4.6405	4.7557	4.1315	3.9484	4.7591
702	Lipids and lipid-like molecules	(3S,5R,6S,7E,9x)-7-Megastigmene-3,6,9-triol 9-glucoside	3.4572	3.4597	4.1738	3.6204	3.8061	3.5046	3.6895	3.5678	3.9111	3.7796
703	Lipids and lipid-like molecules	Methyl (R)-8-Hydroxy-9-decene-4,6-diynoate glucoside	5.1141	4.9997	5.3781	5.0044	5.2554	4.9016	5.2338	5.2526	5.1326	5.1049
704	Lipids and lipid-like molecules	Capsianoside IV	0.4795	0.4474	0.4759	0.4931	0.4906	0.6952	0.4815	0.4397	0.4498	0.475
705	Lipids and lipid-like e	Pantoyllactone glucoside	5.0541	4.15	4.9015	4.8903	4.6293	4.9697	4.8263	4.6871	4.078	4.8931

	molecules											
706	Lipids and lipid-like molecules	Methyl (R)-9-hydroxy-10-undecene-5,7-diynoate glucoside	6.1287	6.2862	5.9339	6.0178	6.1762	6.3328	6.1827	6.0305	6.3843	6.2479
707	Lipids and lipid-like molecules	1-Hexanol arabinosylglucoside	5.0862	5.4856	5.1931	5.0665	5.1658	5.448	5.3111	4.4641	5.6197	5.6981
708	Lipids and lipid-like molecules	Citrusin D	4.7123	4.3388	4.8487	4.9583	4.4641	4.747	4.6108	4.6423	4.5975	4.5104
709	Lipids and lipid-like molecules	1-(3-Methylbutanoyl)-6-apiosylglucose	5.0607	2.9583	2.7194	2.3971	3.2146	3.5168	3.0581	2.854	3.2052	3.1757
710	Lipids and lipid-like molecules	Methyl 7-epi-12-hydroxyjasmonate glucoside	2.8384	2.8937	2.9032	3.2181	2.9672	3.2702	2.8413	2.9474	3.1683	2.8872

711	Lipids and lipid-like molecules	Butyl (S)-3-hydroxybutyrate glucoside	2.6736	2.5505	3.3586	3.0737	2.7044	3.0212	2.7751	2.5861	3.872	2.5926
712	Lipids and lipid-like molecules	Beta-D-Glucopyranosyl-11-hydroxyjasmonic acid	4.4846	5.4066	4.1913	4.5459	4.2405	4.8425	5.2826	4.0874	4.1494	4.3599
713	Lipids and lipid-like molecules	Corchoionol C 9-glucoside	5.2679	3.349	3.3921	4.0649	3.3729	5.526	3.5074	3.0973	5.0587	4.7391
714	Lipids and lipid-like molecules	Blumenol C glucoside	5.0138	3.0634	0.7427	1.9094	0.7406	5.5488	1.1479	0.7975	5.603	4.3853
715	Lipids and lipid-like molecules	Glucosylgalactosyl hydroxylysine	6.029	5.8966	5.9763	5.5867	5.9639	5.8449	6.1363	6.1131	5.7035	5.9367
716	Lipids	1-Acetoxy-2-hydroxy-5,1	6.468	6.4927	6.4388	6.1634	6.3075	6.4756	6.5442	6.4859	6.3398	6.1283

	and lipid-like molecules	2,15-heneicosatrien-4-one										
717	Lipids and lipid-like molecules	Muricatenol	5.9146	5.142	4.9576	5.8082	5.7716	5.2519	5.3077	5.0459	5.3731	5.5192
718	Lipids and lipid-like molecules	Asitrilobin D	5.4266	4.7643	5.045	5.3842	5.3158	4.9603	4.9837	4.8371	4.729	5.1065
719	Lipids and lipid-like molecules	4,6-Heptadiyne-1,3-diol	5.9315	5.9019	5.8618	6.1274	6	6.1445	5.941	5.9369	5.8873	5.916
720	Lipids and lipid-like molecules	4,6-Decadiyn-1-ol	4.5827	4.5654	4.5728	4.5607	4.6117	4.6567	4.5773	4.5486	4.5711	4.7463
721	Lipids and	Porric acid B	5.2858	5.2075	5.6346	5.6332	5.7888	5.3945	5.5459	5.5621	5.5795	5.5732

	lipid-like molecules											
722	Lipids and lipid-like molecules	(3R,7R)-1,3,7-Octanetriol	2.967	2.9322	3.5055	3.0061	2.9797	3.2236	3.0509	2.8965	3.4293	2.9553
723	Lipids and lipid-like molecules	(3Z,6Z)-3,6-Nonadien-1-ol	5.429	5.2885	5.3412	5.4754	5.4532	5.4312	5.366	5.3973	5.2768	5.3981
724	Lipids and lipid-like molecules	Oleyl alcohol	5.3851	5.8436	5.7047	5.5503	5.4928	5.5881	5.7247	5.5212	5.6995	5.5887
725	Lipids and lipid-like molecules	Muricin A	4.8361	2.9921	6.0608	6.3368	5.7731	6.1515	6.1142	5.3101	5.4398	5.9205
726	Lipids and lipid-lik	Squamocin K	6.0803	5.4145	6.7831	7.1352	6.6649	7.168	6.9898	6.4226	6.3483	6.7375

	e molecules										
727	Lipids and lipid-like molecules	13-HDoHE	5.9249	5.2962	5.3565	5.7228	6.0356	5.4751	5.7105	5.6511	5.8281	5.9415
728	Lipids and lipid-like molecules	Dammaradienol	5.9872	5.8022	5.1515	6.3392	6.0965	5.9512	6.0568	6.074	5.8122	5.8556
729	Lipids and lipid-like molecules	Artemoin A	5.4602	5.1184	4.3422	5.3901	5.3113	5.2573	5.1831	5.1085	5.122	5.1576
730	Lipids and lipid-like molecules	4-Hydroxy-6-eicosanone	6.146	5.6517	5.631	6.2745	5.7456	6.1015	5.9295	5.8476	5.696	5.4622
731	Lipids and lipid-like e	1,2,4-Nonadecanetriol	5.765	5.6834	5.6657	5.6334	5.7947	5.6593	5.6979	5.7549	5.699	5.8499

	molecules											
732	Lipids and lipid-like molecules	1,24-Tetracosanediol	5.1235	4.5576	4.9333	5.6406	5.0954	5.2416	5.0821	4.3622	3.6193	4.629
733	Lipids and lipid-like molecules	2-Undecen-1-ol	6.4813	6.3897	6.44	6.3908	6.3817	6.3912	6.37	6.1853	6.2156	6.2776
734	Lipids and lipid-like molecules	4-Hydroxy-6-heneicosanone	4.1671	3.4762	4.1514	3.8154	4.3548	5.6703	3.7634	3.9974	3.5617	3.4901
735	Lipids and lipid-like molecules	(3E,5Z)-3,5-Octadien-1-ol	5.741	5.495	5.4935	5.6451	5.7063	5.7904	5.6636	5.4998	5.4265	5.5946
736	Lipids and lipid-like molecules	Crithmumdiol	5.9344	5.8014	6.2595	5.9615	5.9294	5.7707	5.83	5.9375	5.734	5.8997

737	Lipids and lipid-like molecules	2-Hexen-1-ol	2.7473	3.6227	3.0496	2.9652	2.7091	2.9446	3.0523	2.8562	3.2605	3.1868
738	Lipids and lipid-like molecules	Ethyl 3-hydroxydodecanoate	5.9437	6.3495	6.197	6.0398	5.9427	6.0381	6.1556	5.9236	6.1695	5.9644
739	Lipids and lipid-like molecules	Safynol	4.9425	4.8151	4.6985	4.6962	4.7102	4.7291	4.7449	4.6582	4.9185	5.0094
740	Lipids and lipid-like molecules	Squamostanal A	4.678	4.4779	4.2517	4.445	4.4838	4.6925	4.6868	4.2863	4.2527	4.7505
741	Lipids and lipid-like molecules	3-(Hydroxymethyl)-2-octanone	4.8558	4.7751	4.7519	4.9071	4.9233	4.7274	4.8227	4.7778	4.7739	4.858
742	Lipids	4-Hydroxynonenal	3.9143	3.9957	4.0466	3.8747	4.04	4.15	4.0444	4.1536	4.3801	4.3739

	and lipid-like molecules										
743	Lipids and lipid-like molecules	Cis-Solamin	4.7891	5.2456	4.6544	5.1834	5.1951	5.9988	5.308	4.8465	5.6845	6.3716
744	Lipids and lipid-like molecules	Momordol	3.7393	4.4176	3.0476	2.536	5.2253	4.5678	4.1674	4.8824	5.3546	5.6082
745	Lipids and lipid-like molecules	(R)-2-Hydroxysterculic acid	4.6767	4.9154	3.9269	3.9525	4.0545	4.9796	5.5531	3.8715	3.8942	4.3799
746	Lipids and lipid-like molecules	(E)-3-decen-1-ol	5.4926	5.6058	5.6139	5.8956	5.7095	5.829	5.6581	5.5621	5.3276	5.5614
747	Lipids and	Asitribin	5.0685	5.2124	4.9157	5.1791	4.6941	5.4354	5.1482	4.513	4.6766	5.4168

	lipid-like molecules											
748	Lipids and lipid-like molecules	Cohibin D	4.3857	4.3988	4.207	4.589	4.6086	5.9636	4.8257	4.0469	4.3363	5.3359
749	Lipids and lipid-like molecules	4-Hydroxy-16,18-tritriacontanedione	5.2298	5.2243	4.9368	5.1041	5.0614	5.9071	5.376	5.264	5.0102	5.9335
750	Lipids and lipid-like molecules	20-Tetracosene-1,18-diol	6.1778	6.3502	5.9639	6.6298	6.3823	6.3686	6.2862	6.3207	5.9987	6.2006
751	Lipids and lipid-like molecules	Rollinecin A	4.5124	3.3754	3.5299	5.4111	4.5114	4.2545	4.2264	4.5531	3.5184	3.9094
752	Lipids and lipid-lik	(Z)-3-Methyl-3-decen-1-ol	6.5321	6.6757	6.5178	6.872	6.735	6.7584	6.6504	6.6082	6.3778	6.6379

	e molecules											
753	Lipids and lipid-like molecules	Muricatacin	5.5815	5.6565	5.543	5.6295	5.5619	5.9081	5.9524	5.0663	5.0417	5.72
754	Lipids and lipid-like molecules	Annonisin	3.8265	4.8652	3.6145	3.5382	5.4655	4.3284	4.4687	5.0252	5.4388	5.6174
755	Lipids and lipid-like molecules	Muricin H	4.78	5.6534	4.9482	5.4565	4.8078	5.8433	5.8533	4.4162	5.164	6.0398
756	Lipids and lipid-like molecules	10-Hydroxymyristic acid methyl ester	2.7308	2.682	2.9013	2.9026	2.7473	2.7799	3.5481	2.6699	2.6856	2.7241
757	Lipids and lipid-like	Ethyl 3-hydroxytridecanoate	4.4254	5.1851	4.5242	4.1336	4.647	4.4595	4.2895	3.7926	4.1281	4.2709

	molecules											
758	Lipids and lipid-like molecules	9-Oxoasimicinone	5.4831	4.5101	4.2925	5.4032	5.1934	5.5594	5.0644	4.0336	2.3983	4.0638
759	Lipids and lipid-like molecules	(Z)-6-Tetradecene-1,3-diyne-5,8-diol	5.5691	5.2365	5.1386	5.1248	6.0508	5.7649	5.725	5.2226	4.9266	5.5427
760	Lipids and lipid-like molecules	Avocadene 1-acetate	5.2886	4.9457	4.863	5.0426	5.1514	5.1254	5.1676	5.3611	4.3566	4.5051
761	Lipids and lipid-like molecules	1-Hydroxy-3-nonanone	4.9729	4.7364	5.0575	5.1423	5.0671	5.5431	5.1383	4.76	4.5308	5.1997
762	Lipids and lipid-like molecules	Tetradecanoylcarnitine	5.5823	5.0896	5.2941	6.0278	5.4773	5.9532	5.5226	5.446	4.8849	5.489

763	Lipids and lipid-like molecules	3-Methylglutarylcarnitine	4.5982	4.7137	4.7793	4.9668	4.6991	4.9635	4.5388	4.6393	4.5891	4.5659
764	Lipids and lipid-like molecules	1,2-Anhydridoniveusin	5.3599	6.6047	5.3649	5.3594	5.4527	5.7764	6.5509	5.3762	5.779	5.6392
765	Lipids and lipid-like molecules	Oleoylcarnitine	6.9898	6.4528	6.9434	7.6901	6.6182	6.8498	6.6244	6.3709	6.012	6.5034
766	Lipids and lipid-like molecules	3-hydroxytetradecanoyl carnitine	4.8951	4.7185	4.8466	4.9996	4.7469	4.8232	4.6279	4.5122	4.499	4.6619
767	Lipids and lipid-like molecules	Alpha-linolenyl carnitine	7.4906	7.2611	7.4833	7.8303	7.3878	7.8025	7.4066	7.2044	6.9745	7.2527
768	Lipids	Ethyl	5.8837	5.7574	5.7157	5.8202	5.8605	6.0268	5.881	5.7484	5.7168	5.8112

	and lipid-like molecules	2-hydroxyisovalerate										
769	Lipids and lipid-like molecules	(R)-3-hydroxybutyrylcarnitine	5.174	3.9733	3.8609	2.5251	4.1175	5.1975	4.1907	1.305	5.1508	5.0546
770	Lipids and lipid-like molecules	Hydroxyisovaleroyl carnitine	4.5783	5.0305	4.5699	3.9062	4.29	4.3104	4.8946	3.6848	3.7764	4.2458
771	Lipids and lipid-like molecules	Cis-3-Hexenyl trans-2-hexenoate	4.781	4.7832	4.8907	4.8579	4.8924	5.0902	4.8438	4.8116	4.7332	4.7723
772	Lipids and lipid-like molecules	Ethyl 2-methyl-3,4-pentadienoate	6.3016	6.5121	6.4881	6.3034	6.3465	6.3232	6.3677	6.2712	6.5099	6.2871
773	Lipids and	Cis-5-Tetradecenoylcarnitine	5.2287	4.9762	5.0825	5.624	5.2984	5.5888	5.1858	4.9418	4.7438	5.0733

	lipid-like molecules											
774	Lipids and lipid-like molecules	Retinyl palmitate	4.951	4.4426	5.4474	5.5259	4.6707	4.7201	4.6238	5.2141	4.282	4.6367
775	Lipids and lipid-like molecules	Octadecyl fumarate	5.0654	4.9862	5.044	5.0956	5.0726	5.0608	5.0748	5.0224	5.0365	5.0389
776	Lipids and lipid-like molecules	Propylene glycol stearate	6.2037	5.5494	5.2994	5.7469	6.1304	6.1626	5.9178	5.638	5.0668	5.1678
777	Lipids and lipid-like molecules	Heptadecanoyl carnitine	4.0999	4.0527	4.0622	5.6384	4.1407	4.1777	4.1338	4.0574	4.057	4.1163
778	Lipids and lipid-lik	3-Hydroxyisovalerylcarnitine	4.2045	4.3162	4.4043	4.826	4.5972	5.2463	4.3289	4.2473	4.3806	4.8642

	e molecules											
779	Lipids and lipid-like molecules	Butyryl-L-carnitine	3.8669	4.159	4.6094	4.9788	4.3125	4.8697	4.347	3.2593	3.4578	3.9923
780	Lipids and lipid-like molecules	Hexadecanedioic acid	3.1606	3.0928	4.6616	3.2201	3.4464	3.4975	3.4908	3.2657	2.9649	3.3747
781	Lipids and lipid-like molecules	Sorbitan laurate	6.4141	6.1052	6.4781	6.8373	6.219	6.9502	6.3528	6.028	6.0804	6.2948
782	Lipids and lipid-like molecules	O-malonyl-L-carnitine	5.2003	4.9264	5.4369	5.3	5.1393	5.172	5.1548	5.0895	5.1114	5.3832
783	Lipids and lipid-like	(+/-)-Ethyl 3-acetoxy-2-methylbutyrate	4.3241	4.2884	4.5155	4.5946	4.6219	4.4261	4.2924	4.2533	4.3171	4.3273

	molecules											
784	Lipids and lipid-like molecules	(+/-)-Methyl 5-acetoxyhexanoate	3.637	3.769	4.0443	4.0052	4.1828	4.637	3.8573	3.6503	4.0135	4.5065
785	Lipids and lipid-like molecules	Malonylcarnitine	4.9265	4.2547	4.8412	4.4631	4.3337	4.9116	4.1397	4.2357	4.6737	4.4385
786	Lipids and lipid-like molecules	Neuraminic acid	4.8726	4.7684	5.1872	4.9684	4.781	4.6623	4.8686	4.7566	4.9328	4.9508
787	Lipids and lipid-like molecules	3-hydroxyoctanoyl carnitine	3.4788	3.653	3.2323	3.0339	3.0439	2.6721	3.2445	3.0269	3.594	3.6118
788	Lipids and lipid-like molecules	Isobutyl 2-furanpropionate	4.4337	4.2152	4.397	4.5242	4.7578	4.8653	4.5617	4.4177	4.4159	4.7433

789	Lipids and lipid-like molecules	Ethyl 10-undecenoate	5.0262	4.5342	4.6981	5.4096	5.3366	5.5431	5.0931	5.2371	5.3853	5.5357
790	Lipids and lipid-like molecules	Sorbitan stearate	5.8785	5.0748	4.7101	5.6655	5.1148	5.6021	4.9268	4.8829	4.4266	4.8583
791	Lipids and lipid-like molecules	Sorbitan palmitate	6.6678	5.9125	5.7669	6.6319	5.9692	6.3048	5.8474	5.889	5.3221	5.9884
792	Lipids and lipid-like molecules	Ethyl 9-hexadecenoate	3.6489	3.6	3.6434	3.669	3.6654	4.3811	3.6518	3.5879	3.6037	5.3564
793	Lipids and lipid-like molecules	Hexadecanedioic acid mono-L-carnitine ester	6.1889	5.6683	5.6475	6.1682	6.0341	5.8922	5.8291	5.6379	5.299	5.845
794	Lipids	Methyl	3.8756	3.2784	3.5883	4.1016	3.2904	5.0039	3.4749	3.7877	4.3577	4.2789

	and lipid-like molecules	octynecarboxylate										
795	Lipids and lipid-like molecules	Stearoyllactic acid	4.9	4.8144	5.0973	5.5746	4.866	4.5607	4.6101	4.8081	3.282	4.509
796	Lipids and lipid-like molecules	3-hydroxypentadecanoyl carnitine	4.7476	4.0816	4.2988	4.271	5.3389	4.0352	4.5964	3.9607	2.921	4.0073
797	Lipids and lipid-like molecules	2-Methylbutyroylcarnitine	5.3205	4.3183	3.0706	3.9765	5.0611	4.4791	5.0155	3.676	1.7603	3.1668
798	Lipids and lipid-like molecules	Ethyl isovalerate	2.6334	2.7786	2.5959	2.8955	2.7048	2.5722	2.5139	2.5966	2.5431	2.6409
799	Lipids and	Ethyl (2E,4E,7Z)-Decatrienoat	4.0409	4.0206	3.995	3.9584	3.9958	3.9545	4.0977	3.9446	3.9829	3.9422

	lipid-like molecules	e										
800	Lipids and lipid-like molecules	Diethyl fumarate	2.7254	3.2597	2.9383	3.3746	3.0953	3.0742	3.091	3.1638	3.3664	3.2152
801	Lipids and lipid-like molecules	4-Ethoxy-4-oxobutanoic acid	6.1589	6.0569	6.0657	5.7927	6.1641	6.0681	6.1874	6.0768	6.3988	5.9188
802	Lipids and lipid-like molecules	PA(18:0/18:1(9Z))	6.139	5.8796	5.6886	6.7418	6.075	6.2627	5.9157	5.9952	5.6163	5.8238
803	Lipids and lipid-like molecules	Alpha-Tocotrienol	5.0399	5.0789	5.037	5.2771	5.1684	5.0798	5.1446	5.0477	5.1004	5.0773
804	Lipids and lipid-lik	Plastoquinone 8	6.2057	5.429	5.3801	5.7167	6.2301	6.1018	6.037	6.0173	6.0668	6.3289

	e molecules											
805	Lipids and lipid-like molecules	5C-aglycone	4.4411	3.464	3.5074	3.533	3.5294	3.8161	3.5157	3.4519	4.8118	4.2696
806	Lipids and lipid-like molecules	Vitamin K1 2,3-epoxide	4.3534	4.1459	4.1688	4.3496	4.2135	3.7627	4.7729	3.8688	3.8614	4.0791
807	Lipids and lipid-like molecules	Dexpanthenol	5.0365	4.5623	5.083	4.7077	4.8356	5.1748	4.7594	4.5528	4.4034	4.8134
808	Lipids and lipid-like molecules	Neopellitorine A	2.7248	2.6777	2.6872	2.7605	2.7656	2.8025	2.7587	2.6825	2.6821	2.7412
809	Lipids and lipid-like e	Docosanamide	4.4936	3.5609	3.6176	5.1765	5.004	4.4923	3.8826	3.6742	3.4794	3.8783

	molecules											
810	Lipids and lipid-like molecules	Palmitic amide	6.1693	6.2576	6.2368	6.2681	6.2599	6.291	6.3245	6.2376	6.2711	6.2645
811	Lipids and lipid-like molecules	Pipericine	3.5199	3.0768	3.7448	3.3545	4.2791	3.9007	3.9244	3.4274	3.458	4.2572
812	Lipids and lipid-like molecules	Linoleamide	5.9412	6.2664	6.2063	6.079	6.0212	6.113	6.0352	5.9075	5.9739	5.964
813	Lipids and lipid-like molecules	Herculin	3.2853	3.3413	3.1797	3.5008	3.5869	5.7083	3.4022	3.784	3.6021	4.0885
814	Lipids and lipid-like molecules	S-aminomethyldihydrolipoamide	4.8241	5.0024	4.9716	5.0195	5.3595	5.415	5.2496	4.7672	4.7177	4.9493

815	Lipids and lipid-like molecules	2,4,12-Octadecatrienoic acid isobutylamide	6.7491	5.8843	6.0874	6.5835	6.1664	6.5189	6.0749	5.9141	5.4693	6.1394
816	Lipids and lipid-like molecules	7-Methylinosine	4.1317	3.4565	4.5619	3.7431	3.8296	3.8367	3.2827	2.9774	4.9683	3.2595
817	Lipids and lipid-like molecules	(S)-Hydroxydecanoyl-CoA	5.5575	5.7095	5.6266	5.6955	5.697	5.4725	5.5667	5.7314	5.5648	5.6755
818	Lipids and lipid-like molecules	Neuromedin N (1-4)	5.1635	5.0669	5.3909	5.2275	5.2064	5.1576	5.1156	5.3509	5.2488	5.2569
819	Lipids and lipid-like molecules	3-Oxo-OPC6-CoA	5.63	5.4302	5.541	5.5179	5.729	5.5071	5.5629	5.7402	5.4144	5.4949
820	Lipids	(2E)-Dodecenoyl-CoA	6.3213	6.3337	6.2291	5.8786	6.5936	6.1822	6.189	6.3017	6.004	6.234

	and lipid-like molecules										
821	Lipids and lipid-like molecules	S-(3-Methylbutanoyl)-dihydrolipoamide-E	5.2976	5.1088	5.1408	5.5616	5.1631	5.0789	5.104	5.0222	5.0503	5.2857
822	Lipids and lipid-like molecules	Tridecyl phloretate	4.3072	3.1504	3.9791	5.3235	3.8998	4.5244	3.9204	3.0863	3.0859	3.8867
823	Lipids and lipid-like molecules	(3E,6Z)-Nonadien-1-yl acetate	3.8512	3.8417	3.9214	3.9164	3.7985	3.8893	3.8573	3.8389	3.8009	3.847
824	Lipids and lipid-like molecules	[6]-Gingerdiol 3,5-diacetate	4.7119	4.4889	4.7353	5.1659	4.8152	4.6348	4.7321	4.7579	4.5453	4.7999
825	Lipids and	Dodecyl acetate	4.7016	4.8162	5.1577	4.6215	4.5694	4.4853	4.6804	4.737	4.7212	4.5742

	lipid-like molecules											
826	Lipids and lipid-like molecules	3-Hydroxy-5Z-octenyl acetate	3.3508	2.7425	2.8997	2.9756	4.4473	4.0794	2.9457	4.4763	4.5379	5.4111
827	Lipids and lipid-like molecules	4-Hydroxyproline galactoside	4.8406	4.3137	4.5492	5.2351	4.862	4.8752	5.0571	5.0506	4.2195	4.4264
828	Lipids and lipid-like molecules	Heptadecanal	4.0132	4.707	5.7804	5.6717	4.4345	4.4301	4.6136	4.7537	5.0445	4.3987
829	Lipids and lipid-like molecules	Hexacosanal	4.7428	4.8131	3.9883	5.2801	4.8607	4.9769	4.8087	4.7269	4.5333	4.66
830	Lipids and lipid-lik	DG(16:0/18:1(11Z)/0:0)	6.3473	6.4296	6.2341	6.1765	6.4297	6.3385	6.3702	6.5059	6.2195	6.2436

	e molecules											
831	Lipids and lipid-like molecules	DG(16:0/18:0/0:0)	5.9375	5.7363	5.4739	5.8243	5.8122	5.8179	5.6039	5.5821	5.4573	5.5764
832	Lipids and lipid-like molecules	DG(10:0/16:0/0:0)	4.9364	3.4077	3.6469	4.2256	4.4074	4.8884	3.7871	3.5626	3.9	4.065
833	Lipids and lipid-like molecules	DG(15:0/22:5(7Z,10Z,13Z,16Z,19Z)/0:0)	4.3031	3.3597	2.9144	3.5387	3.822	3.5282	3.6637	3.5724	3.6165	3.5757
834	Lipids and lipid-like molecules	DG(15:0/22:0/0:0)	3.779	3.7248	3.9229	4.1092	3.819	3.9068	4.0612	3.7998	3.7479	3.7117
835	Lipids and lipid-like e	DG(15:0/20:4(8Z,11Z,14Z,17Z)/0:0)	5.6955	6.1671	5.7429	5.8602	5.7452	5.9611	5.9742	5.796	5.9873	5.8236

	molecules										
836	Lipids and lipid-like molecules	DG(20:1(11Z)/18:4(6Z,9Z,12Z,15Z)/0:0)	4.6763	4.1999	4.4919	5.5087	4.7981	4.9132	4.8329	4.4439	4.4602	4.9221
837	Lipids and lipid-like molecules	DG(18:1(11Z)/20:0/0:0)	4.997	5.1906	5.131	5.3288	5.3416	5.2999	5.3673	5.3551	5.2765	5.2956
838	Lipids and lipid-like molecules	DG(20:1(11Z)/16:1(9Z)/0:0)	5.5757	5.4154	5.5656	5.4916	5.4976	5.5205	5.4617	5.3583	5.262	5.4006
839	Lipids and lipid-like molecules	DG(15:0/20:0/0:0)	4.7156	4.6789	4.8576	4.691	4.5725	4.9471	4.7673	4.4537	4.2946	4.5023
840	Lipids and lipid-like molecules	DG(14:0/20:3(5Z,8Z,11Z)/0:0)	4.8301	4.7051	4.9133	5.1934	4.6989	4.7759	4.5631	5.2613	4.5992	4.5409

841	Lipids and lipid-like molecules	DG(18:1(11Z)/14:1(9Z)/0:0)	4.2001	4.3481	4.02	4.0083	4.3825	4.8132	4.2203	4.2969	3.8668	3.9069
842	Lipids and lipid-like molecules	DG(15:0/14:0/0:0)	4.2731	4.4701	4.7038	4.8897	4.8115	5.1628	4.7953	4.8203	4.5187	4.5965
843	Lipids and lipid-like molecules	DG(14:1(9Z)/15:0/0:0)	5.0679	5.1327	5.1046	5.4314	5.2976	5.7988	5.3831	5.3232	5.3207	5.2104
844	Lipids and lipid-like molecules	3-(Acetyloxy)-2-hydroxypropyl icosanoate	5.6576	4.8896	5.4798	6.2849	5.204	5.31	5.0511	4.9271	4.3922	5.1958
845	Lipids and lipid-like molecules	2-Hydroxy-3-methoxyestrone	5.4567	5.0306	5.2031	5.6073	5.0584	5.7594	5.1585	4.8655	4.5192	5.2356
846	Lipids	DG(20:3(5Z,8Z,11Z)/16:1	5.352	4.5317	4.5804	4.2096	5.5763	3.5585	4.8565	4.9508	5.1991	5.9095

	and lipid-like molecules	(9Z)/0:0)										
847	Lipids and lipid-like molecules	3-(Acetyloxy)-2-hydroxypropyl octadecanoate	6.1044	5.5167	5.972	6.799	5.7439	5.7328	5.7027	5.5222	5.2094	5.7093
848	Lipids and lipid-like molecules	3-(2-Heptenyloxy)-2-hydroxypropyl undecanoate	3.4896	3.2573	3.2668	4.0344	3.8465	3.5622	3.5899	3.3415	3.2617	3.6893
849	Lipids and lipid-like molecules	DG(18:4(6Z,9Z,12Z,15Z)/20:1(11Z)/0:0)	6.54	5.6883	6.1509	6.363	6.4921	6.3743	6.3451	6.0599	5.6207	6.175
850	Lipids and lipid-like molecules	DG(20:5(5Z,8Z,11Z,14Z,17Z)/15:0/0:0)	5.2966	5.3986	5.0077	5.1684	4.9289	5.7773	5.3357	5.1215	5.0947	5.7514
851	Lipids and	DG(18:1(11Z)/22:6(4Z,7Z,10Z,13Z,16Z,19Z)/0:0)	3.2885	3.4289	3.7737	4.2558	3.6732	3.515	3.2442	3.558	3.753	3.52

	lipid-like molecules											
852	Lipids and lipid-like molecules	DG(18:1(11Z)/15:0/0:0)	6.1338	6.1595	5.7834	6.0303	5.998	6.9732	6.1518	6.2185	6.0772	6.7733
853	Lipids and lipid-like molecules	DG(16:0/20:0/0:0)	5.2022	4.0655	4.54	5.2017	5.3564	5.0749	5.099	4.497	3.3223	4.7348
854	Lipids and lipid-like molecules	DG(15:0/20:1(11Z)/0:0)	5.2514	4.8871	4.8799	4.9861	4.9337	6.3953	4.823	4.9631	5.1045	5.9725
855	Lipids and lipid-like molecules	DG(15:0/16:1(9Z)/0:0)	5.4275	5.4979	5.185	5.3303	5.2119	5.833	5.6264	5.1897	5.006	5.9394
856	Lipids and lipid-lik	DG(14:0/18:1(11Z)/0:0)	4.5882	4.8229	4.7533	4.4211	4.8079	4.7922	5.2279	3.5918	3.3625	4.5996

	e molecules											
857	Lipids and lipid-like molecules	DG(16:1(9Z)/22:6(4Z,7Z,10Z,13Z,16Z,19Z)/0:0)	3.6194	4.1591	3.5382	3.5803	3.8879	4.4118	4.1753	4.4241	3.457	3.7388
858	Lipids and lipid-like molecules	DG(15:0/17:0/0:0)	3.7809	4.2432	3.7754	3.801	3.7975	5.3514	3.9053	3.72	4.7008	6.2119
859	Lipids and lipid-like molecules	DG(8:0/13:0/0:0)	5.1275	3.4186	3.3543	3.3799	4.1744	3.6133	3.9879	4.1253	3.3146	3.3531
860	Lipids and lipid-like molecules	DG(8:0/0:0/8:0)	2.8027	2.8849	3.2042	3.2596	2.9865	3.3732	3.1215	3.1377	3.3812	3.6824
861	Lipids and lipid-like	(+/-)-Glycerol 1,2-diacetate	4.4134	4.7683	4.7099	4.3558	4.6632	4.7224	4.7905	4.5979	4.7047	4.5225

	molecules											
862	Lipids and lipid-like molecules	DG(16:0e/18:0/0:0)	6.5003	6.5344	6.3624	6.2502	6.3905	6.306	6.414	6.4355	6.3238	6.2057
863	Lipids and lipid-like molecules	DG(15:1(9Z)/18:2(9Z,12Z)/0:0)[iso2]	5.4534	5.5169	5.0046	4.9284	4.9447	6.1652	5.7645	4.5464	4.8175	5.7528
864	Lipids and lipid-like molecules	DGDG(18:3/18:3)	4.2743	4.2254	4.2688	4.2944	4.2908	4.3235	4.2771	4.2133	4.3198	4.2676
865	Lipids and lipid-like molecules	DGDG(16:0/18:3)	3.6011	3.5522	3.5956	3.6212	3.6239	3.6503	3.6039	3.5401	3.5559	3.5944
866	Lipids and lipid-like molecules	DGDG(18:1/18:3)	6.1198	6.1883	6.1607	6.0756	6.2729	5.9514	6.089	6.2578	6.3981	6.0018

867	Lipids and lipid-like molecules	MG(0:0/24:6(6Z,9Z,12Z,15Z,18Z,21Z)/0:0)	4.1725	2.9449	3.4594	4.387	3.5257	3.6598	3.7281	2.2545	2.2541	3.127
868	Lipids and lipid-like molecules	MG(15:0/0:0/0:0)	5.6883	5.7351	5.8778	5.6338	5.8834	5.4756	5.7482	5.6798	5.5587	5.6463
869	Lipids and lipid-like molecules	MG(a-21:0/0:0/0:0)[rac]	3.0582	2.9968	3.0701	3.317	3.2319	3.0569	3.2616	3.3144	3.2521	3.3903
870	Lipids and lipid-like molecules	MG(0:0/22:0/0:0)	5.6265	4.4285	4.1696	5.017	5.1696	4.8498	5.2195	4.5923	3.3472	4.1603
871	Lipids and lipid-like molecules	MG(0:0/14:0/0:0)	5.0868	5.0151	5.0947	5.573	4.8744	5.4737	4.9976	4.8434	4.4281	4.9852
872	Lipids	MG(19:0/0:0/0:0)	5.1642	4.9602	5.2505	5.9509	5.5085	4.8946	4.8953	5.1308	2.5025	4.6288

	and lipid-like molecules											
873	Lipids and lipid-like molecules	MG(0:0/24:0/0:0)	4.1128	4.1583	3.8507	4.1198	4.0303	3.3487	4.6365	3.8796	3.6559	3.5717
874	Lipids and lipid-like molecules	MG(0:0/16:1(9Z)/0:0)	5.587	4.8809	4.6402	4.8932	5.2141	5.1635	5.0542	5.1972	3.5356	4.4806
875	Lipids and lipid-like molecules	MG(0:0/i-12:0/0:0)	4.9374	4.8636	4.487	4.765	4.7097	4.9717	4.8554	4.4083	3.7172	4.3134
876	Lipids and lipid-like molecules	MG(0:0/14:1(9Z)/0:0)	5.637	5.4571	6.187	6.1759	5.5005	6.7486	6.0067	5.8019	4.9574	5.7618
877	Lipids and	TG(18:1/18:2/18:2)	7.5224	7.5583	7.0145	7.4736	7.5001	7.5103	7.4862	7.4314	7.4143	7.4621

	lipid-like molecules											
878	Lipids and lipid-like molecules	TG(18:2/18:2/18:2)	7.3099	7.3316	6.823	7.3676	7.3122	7.1928	7.2338	7.2039	7.0947	7.057
879	Lipids and lipid-like molecules	TG(18:0/20:0/18:4(6Z,9Z,12Z,15Z))	6.3997	6.3403	6.2277	4.7212	6.7258	6.8084	6.6408	5.5899	6.7205	6.6681
880	Lipids and lipid-like molecules	Glycerol 1,3-di-(9Z,12Z-octadecadienoate) 2-(9Z-octadecenoate)	6.7173	6.3661	5.718	6.325	6.5069	6.4521	6.3674	6.2945	6.3074	6.2709
881	Lipids and lipid-like molecules	TG(15:0/18:2(9Z,12Z)/22:6(4Z,7Z,10Z,13Z,16Z,19Z))	6.228	5.7324	4.5255	6.3967	6.0443	5.9624	5.9394	5.8116	5.6816	5.8365
882	Lipids and lipid-lik	TG(18:3/18:3/18:3)	3.9765	3.3902	2.7195	3.659	3.5683	3.4099	3.4916	3.0418	2.8656	3.2298

	e molecules											
883	Lipids and lipid-like molecules	TG(18:2(9Z,12Z)/18:2(9Z,12Z)/18:2(9Z,12Z))	6.2572	5.8679	4.9977	5.989	6.0988	5.9563	5.9273	5.7346	5.5804	5.6986
884	Lipids and lipid-like molecules	TG(8:0/8:0/15:0)	5.4914	5.8573	5.717	5.6682	5.6736	6.2008	6.3146	5.317	5.3873	5.8989
885	Lipids and lipid-like molecules	N-Methyl-14-O-demethylepiporphyroxine	4.8913	4.639	4.2133	4.8882	4.4909	3.9466	4.8018	4.1515	4.2862	4.504
886	Lipids and lipid-like molecules	2,3-Diacetoxypropyl stearate	5.2787	5.2422	5.5712	5.9898	5.2863	6.0438	5.3919	5.1157	5.0428	5.5389
887	Lipids and lipid-like	Gingerglycolipid B	5.8596	4.7128	4.202	5.2471	5.0241	4.5753	4.5423	4.8054	4.3564	4.832

	molecules											
888	Lipids and lipid-like molecules	Galactosylglycerol	5.1756	4.7371	4.8249	4.5071	4.8333	4.9769	4.6006	5.0731	4.8972	5.2019
889	Lipids and lipid-like molecules	Gingerglycolipid A	2.8857	2.8368	2.8802	2.9058	2.9022	2.9348	2.8885	2.8248	2.8405	2.879
890	Lipids and lipid-like molecules	N-Stearoylsphingosine	6.1943	5.7196	6.089	6.4898	6.0272	6.1062	5.8431	5.6779	5.2969	5.9714
891	Lipids and lipid-like molecules	N-[(4E,8E)-1,3-dihydroxyoctadeca-4,8-dien-2-yl]hexadecanamide	5.4182	5.6777	5.5509	5.463	5.465	5.28	5.3621	5.6298	5.325	5.2548
892	Lipids and lipid-like molecules	Ceramide (d18:1/12:0)	5.666	4.9851	4.9547	5.5013	4.8976	5.3274	5.1033	5.5991	4.1092	4.8301

893	Lipids and lipid-like molecules	Cer(d18:1/18:1(11Z))	5.5415	4.4437	4.9425	5.4271	5.3731	5.2518	5.1031	5.3257	3.7337	4.7489
894	Lipids and lipid-like molecules	Cer(d18:1/18:0)	5.2808	4.0802	3.8993	5.2699	5.298	5.0371	4.827	4.4328	3.5127	3.6615
895	Lipids and lipid-like molecules	Ceramide (d18:1/9Z-18:1)	5.3906	4.8207	4.9252	5.6537	5.2256	5.1954	4.9772	4.6833	4.1937	5.0568
896	Lipids and lipid-like molecules	Cer(d18:0/18:0)	4.7008	3.7558	3.9253	5.3414	4.8896	4.8989	4.6247	4.0607	3.4737	4.7935
897	Lipids and lipid-like molecules	Cer(d18:0/16:0)										
